# Sex-specific body fat distribution predicts cardiovascular ageing

**DOI:** 10.1093/eurheartj/ehaf553

**Published:** 2025-08-22

**Authors:** Vladimir Losev, Chang Lu, Shamin Tahasildar, Deva S Senevirathne, Paolo Inglese, Wenjia Bai, Andrew P King, Mit Shah, Antonio de Marvao, Declan P O’Regan

**Affiliations:** MRC Laboratory of Medical Sciences, Imperial College London, Hammersmith Hospital Campus, London, UK; MRC Laboratory of Medical Sciences, Imperial College London, Hammersmith Hospital Campus, London, UK; MRC Laboratory of Medical Sciences, Imperial College London, Hammersmith Hospital Campus, London, UK; MRC Laboratory of Medical Sciences, Imperial College London, Hammersmith Hospital Campus, London, UK; MRC Laboratory of Medical Sciences, Imperial College London, Hammersmith Hospital Campus, London, UK; Department of Computing, Imperial College London, London, UK; Department of Brain Sciences, Imperial College London, London, UK; School of Biomedical Engineering & Imaging Sciences, King’s College London, London, UK; MRC Laboratory of Medical Sciences, Imperial College London, Hammersmith Hospital Campus, London, UK; MRC Laboratory of Medical Sciences, Imperial College London, Hammersmith Hospital Campus, London, UK; Department of Women and Children’s Health, King’s College London, London, UK; British Heart Foundation Centre of Research Excellence, School of Cardiovascular and Metabolic Medicine and Sciences, King’s College London, London, UK; MRC Laboratory of Medical Sciences, Imperial College London, Hammersmith Hospital Campus, London, UK

**Keywords:** Ageing, Body composition, Sex differences, MRI

## Abstract

**Background and Aims:**

Cardiovascular ageing is a progressive loss of physiological reserve, modified by environmental and genetic risk factors, that contributes to multi-morbidity due to accumulated damage across diverse cell types, tissues, and organs. Obesity is implicated in premature ageing, but the effect of body fat distribution in humans is unknown. This study determined the influence of sex-dependent fat phenotypes on human cardiovascular ageing.

**Methods:**

Data from 21 241 participants in the UK Biobank were analysed. Machine learning was used to predict cardiovascular age from 126 image-derived traits of vascular function, cardiac motion, and myocardial fibrosis. An age-delta was calculated as the difference between predicted age and chronological age. The volume and distribution of body fat was assessed from whole-body imaging. The association between fat phenotypes and cardiovascular age-delta was assessed using multivariable linear regression with age and sex as co-covariates, reporting β coefficients with 95% confidence intervals (CI). Two-sample Mendelian randomization was used to assess causal associations.

**Results:**

Visceral adipose tissue volume [β = 0.656, (95% CI, .537–.775), *P* < .0001], muscle adipose tissue infiltration [β = 0.183, (95% CI, .122–.244), *P* = .0003], and liver fat fraction [β = 1.066, (95% CI .835–1.298), *P* < .0001] were the strongest predictors of increased cardiovascular age-delta for both sexes. Abdominal subcutaneous adipose tissue volume [β = 0.432, (95% CI, .269–.596), *P* < .0001] and android fat mass [β = 0.983, (95% CI, .64–1.326), *P* < .0001] were each associated with increased age-delta only in males. Genetically predicted gynoid fat showed an association with decreased age-delta.

**Conclusions:**

Shared and sex-specific patterns of body fat are associated with both protective and harmful changes in cardiovascular ageing, highlighting adipose tissue distribution and function as a key target for interventions to extend healthy lifespan.

## Structured Graphical Abstract

**Structured Graphical Abstract:**
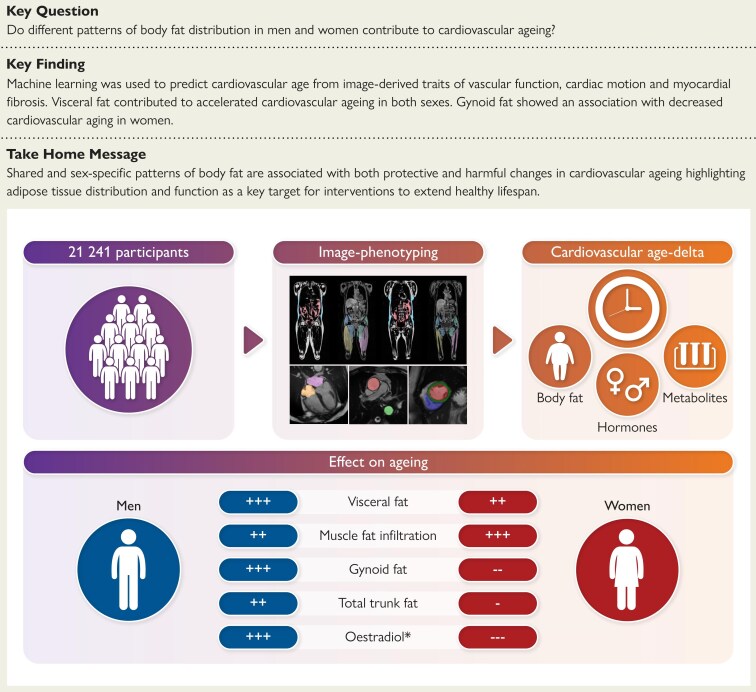
The association of body fat phenotypes and cardiovascular ageing was assessed in 21 241 participants. This showed how shared and sex-specific patterns of body fat are associated with protective and harmful changes in cardiovascular ageing. *Protective effects of oestradiol in pre-menopausal women.

**See the editorial comment for this article ‘Sex- and depot-specific adiposity as a driver of cardiovascular ageing’, by P.-G. Masci and S.E. Petersen, https://doi.org/10.1093/eurheartj/ehaf554.**

## Introduction

Obesity is a chronic complex condition characterized by excessive fat deposits that are detrimental to health. Globally, 43% of adults, affecting men and women in equal proportions, are overweight, with a rising prevalence.^1^ It is a multifactorial and heterogeneous disease related to obesogenic environments, psycho-social influences, and genetic risk factors. Individuals with similar body mass index (BMI) may have distinct metabolic and cardiovascular disease risk profiles; therefore, susceptibility to obesity-related cardiovascular complications is not mediated solely by overall body mass but may be strongly influenced by variation in fat distribution.^2^ For instance, visceral fat confers a higher risk of adverse outcomes,^3^ promotes systemic and vascular inflammation,^4,5^ and is independently linked with dysmetabolic profiles.^6^

Obesity, as a systemic syndrome of adipose tissue dysfunction, can be considered a pro-inflammatory process of accelerated cardiovascular ageing with which it shares common genetic, epigenetic, immunological, and metabolic mechanisms.^7,8^ Recently, imaging and physiological parameters have been used to assess environmental and genetic risk factors for accelerated biological ageing, which point to complex inter-organ ageing networks that are regulated by genes involved in inflammation, tissue elasticity, and pro-fibrotic pathways.^9,10^ Such approaches use machine learning to predict the deviation from healthy ageing across multiple image-derived phenotypes in the heart and circulation. Less is known about drivers of sexual dimorphism in cardiovascular ageing, although cellular and molecular mechanisms of ageing may be better maintained in women until the menopause.^11^ Sex hormones modify key aspects of nutrient sensing, metabolism, and fat depot regulation—with men tending to have more visceral fat, whereas women have greater fat deposition in the lower body.^12^ The role of sex-specific fat distribution on accelerated cardiovascular ageing is not known, but could be a key modifiable mediator of obesity-related risk.

In this study, we use machine learning techniques trained to predict biological age from multiple image-derived cardiovascular traits that assess the structure, function and tissue characteristics of the heart and circulation.^9^ We then express how an individual’s cardiovascular system has aged relative to a normative population using an ‘age-delta’. Through assessing body fat distributions with whole-body image phenotyping, as well as circulating biomarkers and sex hormones, we aimed to understand the relationship between fat distribution and cardiovascular ageing in over 20 000 middle-aged men and women.

## Methods

### Study overview

The UK Biobank comprises approximately 500 000 community-dwelling participants aged 40–69 years who were recruited across the United Kingdom between 2006 and 2010.^13^ All participants provided written informed consent for participation in the study, which was approved by the National Research Ethics Service (11/NW/0382). We included participants with prevalent disease, including cardiovascular disease or events, to reflect the lifetime cumulative effects of ageing risk exposures. Our study was conducted under terms of access approval number 40 616.

**Figure 1:**
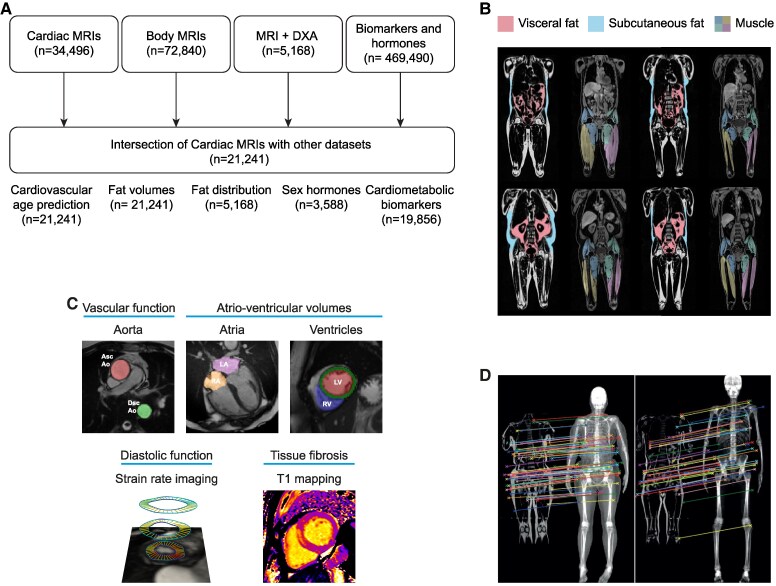
Analysis of fat phenotypes and cardiovascular ageing. *A*) Flowchart of analyses performed in UK Biobank participants. *B*) Fat phenotyping was performed by segmentation of whole-body MRI into visceral, subcutaneous, and muscle compartments (credit: AMRA Medical). *C*) Phenotypes derived from cardiac MRI were used for age prediction. These included automated time-resolved segmentations of the aorta and cardiac chambers, as well as strain rate analysis and T1 mapping. *D*) Integration of MRI and DXA-enabled regional body composition analysis (credit: Rhydian Windsor^14^). MRI, magnetic resonance imaging; DXA, dual X-ray absorptiometry; Asc Ao, ascending aorta; Dsc Ao, descending aorta.

An outline of the methods is shown in *Figure 1*. We used a pre-trained model to predict cardiovascular age from 126 image-derived traits of vascular function, cardiac motion, and myocardial fibrosis. Cardiovascular age-delta was calculated as the difference between predicted age and chronological age.^9^ We combined data from body imaging to assess phenotypes of fat volume and distribution, including visceral adipose tissue, abdominal subcutaneous adipose tissue, muscle adipose tissue infiltration, liver proton density fat fraction, total abdominal adipose tissue, android adipose tissue mass, and gynoid adipose tissue mass, as well as total trunk and whole-body fat mass.

We used multivariable linear regression modelling stratified by sex to assess the association of fat phenotypes with cardiovascular ageing. Additionally, the relationship between BMI categories and fat phenotypes by sex was assessed.

### Cardiac image acquisition

A standardized cardiac magnetic resonance (CMR) imaging protocol was followed to acquire two-dimensional, retrospectively gated cine imaging on a Siemens MAGNETOM Aera 1.5T scanner (Siemens Healthineers, Erlangen, Germany).^15^ Short-axis cine imaging comprised a contiguous stack of images from the left ventricular base to apex, and long-axis cine imaging was performed in the two- and four-chamber planes. Cine sequences consisted of 50 cardiac phases with an average temporal resolution of 31 ms.^15^ Transverse cine imaging of the ascending and descending thoracic aorta was also performed. Native T1 mapping within a single breath-hold was performed at mid-ventricular level using a shortened modified Look-Locker inversion recovery (ShMOLLI) sequence. Imaging phenotypes all underwent quality control prior to use in analysis.^16^

### Cardiac image analysis

Automated segmentation of the short-axis and long-axis cine images in the UK Biobank was performed using fully convolutional networks.^17^ Volumes (end-diastolic, end-systolic, and stroke volume) and ejection fraction were determined for both ventricles. Myocardial volumes were used to compute left ventricular myocardial mass, assuming a density of 1.05 g.ml^−1^. Atrial volumes were calculated using the biplane area–length method.^18^ Central vascular function was assessed by measuring aortic distensibility from central blood pressure estimates and dynamic aortic imaging.^18^ The aorta was segmented on the cine images with a spatiotemporal neural network,^19^ from which maximum and minimum cross-sectional areas were derived. Distensibility was calculated using central blood pressure estimates obtained using peripheral pulse-wave analysis (Vicorder, Wuerzburg, Germany).^18^

Diastolic function, which is a key feature of the ageing heart, was assessed using motion analysis to derive end-diastolic strain rates.^17^ Non-rigid image registration between successive frames enabled motion tracking on greyscale images.^20^ Registration was performed in both backwards and forwards directions from end-diastole, resulting in an averaged displacement field,^18^ which was then used to warp segmentations from end-diastole to successive adjacent frames. Circumferential (E_cc_) and radial (E_rr_) strains were calculated using short-axis cines. Longitudinal (E_ll_) strain was calculated from long-axis four-chamber motion tracking measured at basal, mid-ventricular, and apical levels. Segmental and global peak strains were then calculated. Strain rate was computed as the first derivative of strain, and thereafter, peak early diastolic strain rate in radial (PDSR_rr_) and longitudinal (PDSR_ll_) directions was calculated. We assessed diffuse myocardial fibrosis, an early feature of natural ageing,^21^ using native T1 mapping of the interventricular septum.^22^ The ShMOLLI T1 maps were analysed using probabilistic hierarchical segmentation with automated quality control, defining a region of interest within the interventricular septum as previously validated.^22^ Blood pool T1 was used as a linear correction of myocardial T1 values.^22,23^ In total, 126 quantitative cardiac imaging phenotypes characterizing structure, function, and tissue characteristics were generated for each participant.

### Cardiovascular age prediction

We used a model to predict cardiovascular age from image-derived phenotypes that was pre-trained on 5065 healthy individuals, not included in the current study, who were free of cardiac, metabolic, or respiratory disease and had a BMI <30 kg/m^2^. The development and performance of the model has been previously reported, and a full description is given in Supplementary Methods.^9^ Briefly, we used CatBoost, a machine learning algorithm based on decision trees and gradient boosting, to estimate the age of each participant from a joint analysis of all cardiac image-derived phenotypes. We randomly split this group into separate training (80%) and test (20%) sets, with a validation holdout subset used for hyperparameter search. Similar to brain age modelling, we addressed the correlation between age-delta and chronological age using a linear regression analysis between the initial unadjusted cardiovascular age-delta and chronological age. Subsequently, an offset was determined by multiplying the chronological age by the slope of the regression line and adding the intercept. This offset was then subtracted from the initial unadjusted predicted age to yield the corrected predicted age.^9^

The trained model is independently predictive of outcomes and age-related diseases, reflects causal aspects of ageing, and is associated with putative therapeutic modifiers of ageing.^9^ In validation studies, it provides a stable estimate of age-delta in different sub-populations. As such, it provides a robust biomarker of differential ageing and changes in response to exposures.^24^

### Body adipose tissue analysis

Abdominal and body adipose tissue assessment was performed on the same 1.5T scanner using a dual-echo Dixon Vibe imaging protocol, enabling a comprehensive evaluation of the entire body from the neck to the knees.^25^ This imaging protocol generated a dataset with water and fat components separated, facilitating the analysis of body fat composition. In brief, six overlapping sections were acquired that underwent calibration, stacking, fusion, and segmentation. Body composition analyses were carried out using AMRA Researcher (AMRA Medical AB, Linköping, Sweden). Values for android and gynoid adipose tissue mass were acquired from over 20 000 subjects in the UK Biobank dataset with multi sequence magnetic resonance and dual-energy X-ray absorptiometry (DXA) scans, using self-supervised, multi-modal alignment for whole-body medical imaging, and transferring segmentation maps from DXA to MRI scans achieving high accuracy in matching different modality scans without requiring ground-truth magnetic resonance examples.^14^

We also utilized datasets for liver proton density fat fraction employing the gradient echo protocol from the UK Biobank, specifically the LMS (Liver MultiScan) Dixon method. The LMS Dixon proton density fat fraction captures images during a single breath-hold, reducing motion artefacts and ensuring consistent measurements. Analysis involves three 15-mm circular regions of interest in the liver parenchyma, calculating proton density fat fraction, liver iron concentration, and iron-corrected T1 (cT1). Proton density fat fraction, a reliable measure of liver fat, is determined using water-fat separation masks, with values over 5% indicating fatty liver disease.^26^

Standing height without shoes was assessed using a Seca (Hamburg, Germany) 202 height measure. Weight, without shoes or outer clothing, was assessed with a Tanita (Tokyo, Japan) body composition analyser. Each BMI was calculated as weight in kilograms divided by height in metres^2^.

### Circulating biomarkers

We assessed nine cardiometabolic and endocrine circulating biomarkers [triglycerides, direct low-density lipoprotein (LDL) cholesterol, high-density lipoprotein (HDL) cholesterol, cholesterol (total), apolipoproteins A and B, sex hormone binding globulin (SHBG), oestradiol (E2), and free testosterone] as potential modifiers of cardiovascular ageing. The collection of these biomarkers involved standardized venous blood sampling followed by serum separation and storage at −80°C to ensure stability. The samples were then analysed using validated biochemical assays to quantify levels of each biomarker.^27^ We also assessed lipid and metabolic biomarkers quantified by nuclear magnetic resonance (NMR) spectroscopy taken at baseline that were generated by Nightingale Health.^28^ A total of 251 metabolites were identified, of which 170 absolute measures were used here. Following normalization and standardization, a least absolute shrinkage and selection operator (LASSO) model was used to select 37 representative metabolite features in 22 534 participants with an age-delta.^29^

### Outcome analysis

For assessing the association of cardiovascular age-delta with prospective cardiovascular events, we evaluated incident composite and component outcomes subsequent to the CMR visit that included major adverse cardiovascular events (MACE), hypertension, atrial fibrillation, stroke, angina, heart failure, myocardial infarction (MI), and type 2 diabetes. Events were identified using International Classification of Diseases (ICD) coding from electronic health records, with the specific ICD-9/10 codes and definitions detailed in Supplementary data online, *Table S1*.

### Physical activity analysis

Physical activity was assessed using metabolic equivalent of task (MET) scores derived from self-reported questionnaires performed according to International Physical Activity Questionnaire (IPAQ) guidelines. MET measures energy expenditure, with higher values reflecting greater activity levels. Total MET-minutes per week were calculated from reported walking, moderate, and vigorous activities, offering a standardized estimate of physical activity, and at least 1200 MET-min/week was used to define highly active participants.^30^

### Mendelian randomization

Mendelian randomization (MR) was performed to investigate the potential causality of adipose tissue volumes on cardiovascular age-delta. Genetic instruments relied on summary statistics from two previously published large-scale genome-wide association studies in the UK Biobank of (i) image-derived visceral, abdominal subcutaneous, and gluteofemoral adipose tissue volumes after adjustment with BMI and height,^31^ and (ii) cardiovascular age-delta.^9^

We used the R package TwoSampleMR to perform the analysis.^32^ Independent genetic instruments were selected at the conventional genome-wide significance threshold of *P* < 5 × 10^−8^, using the 1000G genomes European population as reference for linkage disequilibrium (LD) with an *r*^2^ threshold of 0.001. Exposure and outcome statistics were harmonized to allow consistency of alleles for MR analysis. Palindromic single-nucleotide polymorphisms (SNPs) with intermediate allele frequencies were removed before MR. For each analysis, we performed MR with five methods,^32^ including the inverse variance-weighted method that assumes all genetic variants are valid IVs, the MR Egger regression as a check for horizontal pleiotropy, the weighted median method, which is additionally robust in the presence of outliers, the simple mode and weighted mode methods which clusters SNPs into groups based on similarity of causal effects.

### Statistics

Analysis was performed in R (version 4.2.3) and Python. Variables were expressed as percentages and frequencies if categorical, mean ± standard deviation (SD) if continuous and normal, and median (interquartile range, IQR) if continuous and non-normal. Comparison of independent groups was performed with a two-sample *t-*test. Multivariable linear regression was used to estimate the association between age-delta (as the dependent variable), and adiposity phenotypes and cardiometabolic biomarkers (as independent variables) using age, age,^2^ and sex as covariates. Additionally, the model was adjusted for height^2^ to account for variation in stature (see Supplementary Methods).^33^ Standardized β coefficients are reported for continuous predictors, each with 95% confidence intervals (CI). The effect of sex on the relationship between fat phenotypes and age-delta was assessed as an interaction in the regression model. Fat mass was categorized using centile ranges of each BMI group and then compared with alluvial plots (see Supplementary Methods and Supplementary data online, *Figure S1*). Time-to-first event analysis of outcome endpoints associated with age-delta was conducted using Cox proportional hazards models adjusted for age and sex. Tukey’s Honestly Significant Difference (HSD) test was used to compare pairwise differences between activity categories while controlling the probability of making one or more Type I errors.

Statistical significance was considered at *P* ≤ .05 (two-sided). The Benjamini–Hochberg procedure was used to control the false discovery rate.

## Results

### Sex-dependent associations of body fat with chronological age

**Figure 2:**
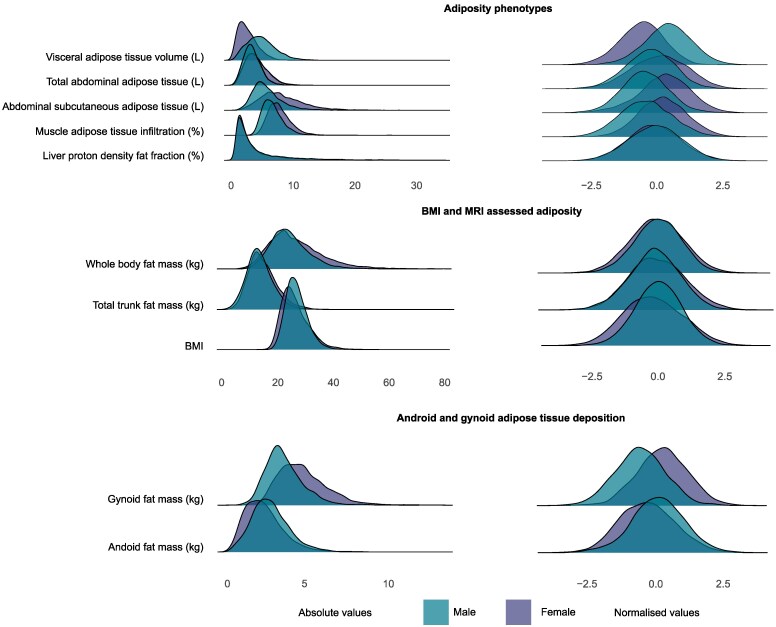
Distribution of fat phenotypes. Ridge plots summarizing the distribution densities of adiposity phenotypes. Unadjusted and normalized values shown. Body mass index, BMI; Magnetic resonance imaging, MRI; Adiposity phenotypes *n* = 21 241; BMI and MRI assessed adiposity *n* = 21 241; Android and gynoid adipose tissue deposition *n* = 5168

**Figure 3:**
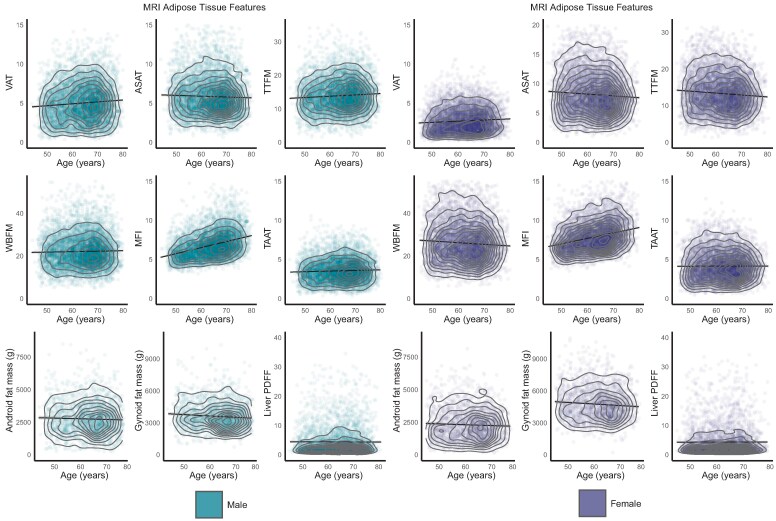
Adiposity associations with chronological age. A selection of representative phenotypes grouped by MRI-derived adipose features (*n* = 21 241), android and gynoid fat mass (*n* = 5168) are shown with their relationship to chronological age at the time of imaging (ages jittered, density contours, point colours represent coefficient of determination (*R*^2^). VAT, visceral adipose tissue; ASAT, abdominal subcutaneous adipose tissue; TTFM, total trunk fat mass; WBFM, whole-body fat mass; MATI, muscle adipose tissue infiltration; TAAT, total abdominal adipose tissue; and PDFF, proton density fat fraction (of the liver)

In total 21 241 participants were included in the analysis with tabular data and age-dependent effects shown in *Table 1* and *Figure 2*. Females had more abdominal subcutaneous fat (8.3 ± 3.5 L vs. 6.0 ± 2.5 L, *P* < .0001), muscle adipose tissue infiltration (7.9 ± 1.9% vs. 6.9 ± 1.7%, *P* < .0001), and gynoid fat (4.8 ± 1.6 kg vs. 3.6 ± 1.2 kg, *P* = .0001), whereas males predominantly had greater volumes of visceral fat (5.1 ± 2.3 L vs. 2.8 ± 1.5 L, *P* < .0001), android fat (2.8 ± 1.2 kg vs. 2.3 ± 1.2 kg, *P* = .0002), and whole-body fat mass (23.9 ± 8.6 kg vs. 20.1 ± 7.0 kg, *P* < .0001). Muscle adipose tissue infiltration increased by an average of 11.7% per decade in females and 9.8% in males. Visceral fat increased more with age in males, rising by an average of 8.2% each decade compared to 5.3% in females. Abdominal subcutaneous fat showed a modest decrease of 5.4% in females and 4.3% in males per decade (*Figure 3*). Adipose phenotype distribution by ancestry is shown in Supplementary data online, *Figure S2* and Supplementary data online, *Table S2*.

**Table 1:** Study population characteristics and baseline cardiac magnetic resonance imaging and adipose tissue parameters by sex

	Female (*n* = 10 558)	Male (*n* = 10 683)
**Baseline characteristics**		
Age at MRI (years)	62.5 ± 7.3	63.9 ± 7.6
Age at menopause (years)	53.6 ± 3.3	-
White ancestry	9690 (91.7)	9822 (91.9)
Black ancestry	181 (1.7)	226 (2.1)
Asian ancestry	386 (3.7)	296 (2.8)
Mixed ancestry	301 (2.9)	339 (3.2)
Body mass index (kg/m^2^)	26.6 ± 4.9	27.3 ± 3.9
Systolic blood pressure (mmHg)	131 ± 17	139 ± 18
Diastolic blood pressure (mmHg)	77 ± 8	82 ± 8
Pulse rate (bpm)	75 ± 7	71 ± 8
Diabetes mellitus	745 (7.1)	1088 (10.2)
Hypercholesterolaemia	975 (9.2)	1749 (16.3)
Hypertension	2129 (20.2)	3082 (28.8)
Coronary artery disease	383 (3.6)	1162 (10.8)
Obesity	2491 (23.6)	2826 (26.5)
Had menopause	8206 (77.7)	-
Current smoker	383 (3.6)	508 (4.8)
Current alcohol consumption	338 (3.2)	483 (4.5)
**Cardiac parameters from CMR**		
Left ventricular ejection fraction (%)	60.7 ± 5.7	58.2 ± 6.2
Left ventricular end-diastolic volume (ml)	133.6 ± 26.5	164.3 ± 33.6
Left ventricular end-systolic volume (ml)	52.8 ± 14.9	69.2 ± 20.2
Left ventricular stroke volume (ml)	80.8 ± 16.0	95.1 ± 19.7
Left ventricular cardiac output (L/min)	5.1 ± 1.1	5.9 ± 1.3
Left ventricular mass (g)	74.5 ± 16.1	100.0 ± 20.3
Right ventricular ejection fraction (%)	58.8 ± 5.7	55.7 ± 6.1
Right ventricular end-diastolic volume (ml)	138.9 ± 29.3	175.6 ± 35.5
Right ventricular end-systolic volume (ml)	57.6 ± 16.3	78.2 ± 20.6
Right ventricular stroke volume (ml)	81.3 ± 17.0	97.3 ± 20.5
**Adiposity parameters from MRI**		
Visceral adipose tissue (L)	2.76 ± 1.5	5.1 ± 2.3
Abdominal subcutaneous adipose tissue (L)	8.3 ± 3.5	6.0 ± 2.5
Muscle adipose tissue infiltration (%)	7.9 ± 1.9	6.9 ± 1.7
Liver proton density fat fraction (%)	4.8 ± 4.3	4.6 ± 4.4
Whole-body fat mass (kg)	20.1 ± 7.0	23.9 ± 8.6
Total trunk fat mass (kg)	14.4 ± 5.0	12.0 ± 4.8
Gynoid fat mass (kg)	4.8 ± 1.6	3.6 ± 1.2
Android fat mass (kg)	2.3 ± 1.2	2.8 ± 1.2

Data are presented as mean ± standard deviation, or *n* (%).

### Association of cardiovascular age-delta and body fat phenotypes

**Figure 4:**
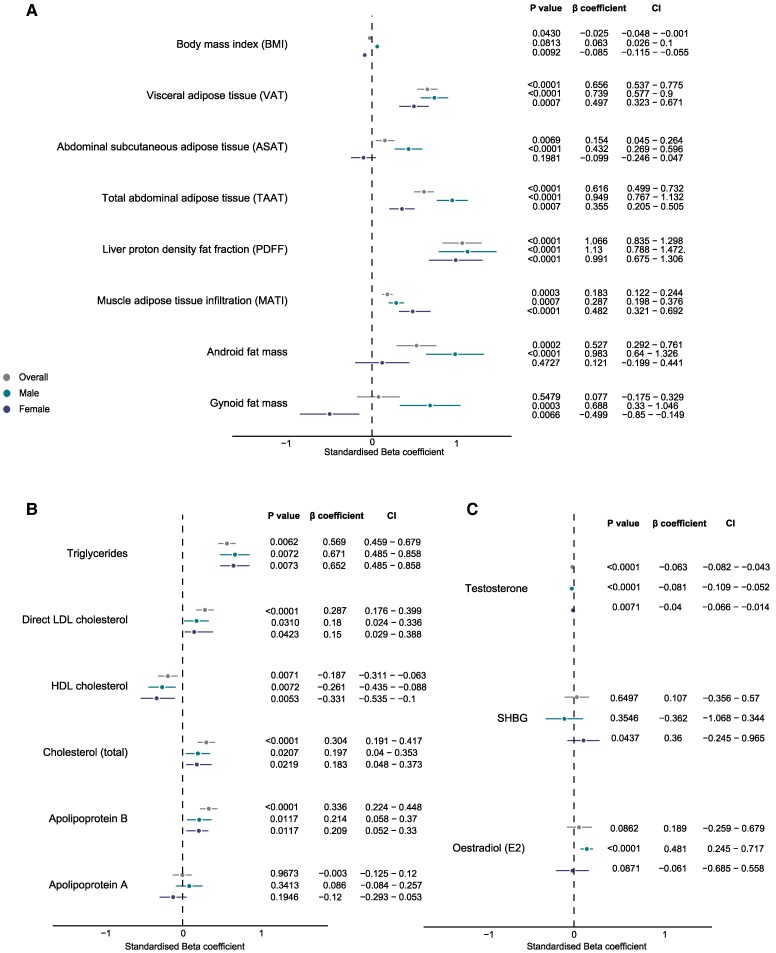
Adiposity phenotype, cardiometabolic and endocrine associations with cardiovascular age-delta. *A*) Linear regression analysis of quantitative adipose tissue traits (*n* = 21,241, of which 5168 had android and gynoid fat mass values) with cardiovascular age-delta as the dependent variable. *P* values, standardized beta-coefficient point estimates, and 95% confidence intervals shown stratified by sex. Linear regression analysis of *B*) circulating lipids (*n* = 19 856) and *C*) sex hormones (*n* = 3588) with cardiovascular age-delta as the dependent variable. *P* values, standardized beta-coefficient point estimates, and 95% confidence intervals shown stratified by sex. LDL, low-density lipoprotein; HDL, high-density lipoprotein; SHBG, sex hormone–binding globulin

Visceral adipose tissue, muscle adipose tissue infiltration, liver fat fraction, and total abdominal adipose tissue were associated with adverse changes in cardiovascular age-delta for both sexes (all *P* ≤ .0005) shown in *Figure 4A*. For males, an increased age-delta was associated with android and gynoid fat. Gynoid fat was significantly associated with beneficial changes in cardiovascular age-delta in pre-menopausal females (*P* = .0011), while this association was not significant in postmenopausal females (*P* = .441). Total trunk fat mass (*P* = .0061) and whole-body fat mass (*P* = .0043) remained associated with beneficial changes in cardiovascular age-delta for all-age females (see Supplementary data online, *Table S3*). BMI was a significant but weaker predictor of age-delta than body fat in both sexes. We found that the relationships between age-delta and fat phenotypes were dependent on sex (interaction results shown in Supplementary data online, *Table S4*). In summary, visceral adipose tissue showed a stronger positive association with age-delta in males (*P* < .0001), while abdominal subcutaneous adipose tissue (*P* = .0001) and trunk fat mass (*P* < .0001) had different directions of effect between males and females.

A diagnosis of diabetes was positively associated with age-delta [β = 3.428, (95% CI, .646–6.210), *P* = .016] and increased the adverse relationship between fat phenotypes and age-delta, including visceral adipose tissue (see Supplementary data online, *Table S5*). In diabetic participants taking Biguanides (metformin), the relationship held, but the effect size on ageing was less [β = 0.481, (95% CI, .070–.893), *P* = .022] (see Supplementary data online, *Table S5*).

The relationship between fat distribution and cardiovascular age-delta remained mainly independent of height^2^ with visceral adipose tissue, liver fat fraction, muscle adipose tissue infiltration and total abdominal adipose tissue (all *P* ≤ .0078) remaining the strongest predictors for increased age-delta overall. Analyses stratified by sex are shown in Supplementary data online, *Figure S3*.

### Mendelian randomization analysis

Using 17, 14, and 25 genetically independent instruments, we tested the causal association of image-derived fat phenotypes with cardiovascular age-delta (see Supplementary data online, *Table S6*). We observed genetic associations of gluteofemoral adipose tissue to cardiovascular age-delta [β = −0.96, (95% CI, −.39 to −1.52), *P* = 8.7 × 10^−4^] from inverse variance-weighted two-sample MR, suggesting a potentially protective role against increasing cardiovascular age-delta. Two other fat traits, visceral and abdominal subcutaneous adipose tissue, did not have a significant association with age-delta but had the opposite effect direction to gluteofemoral adipose tissue (see Supplementary data online, *Table S7* and Supplementary data online, *Figure S4*).

### Relationship between circulating biomarkers and cardiovascular age-delta

**Figure 5:**
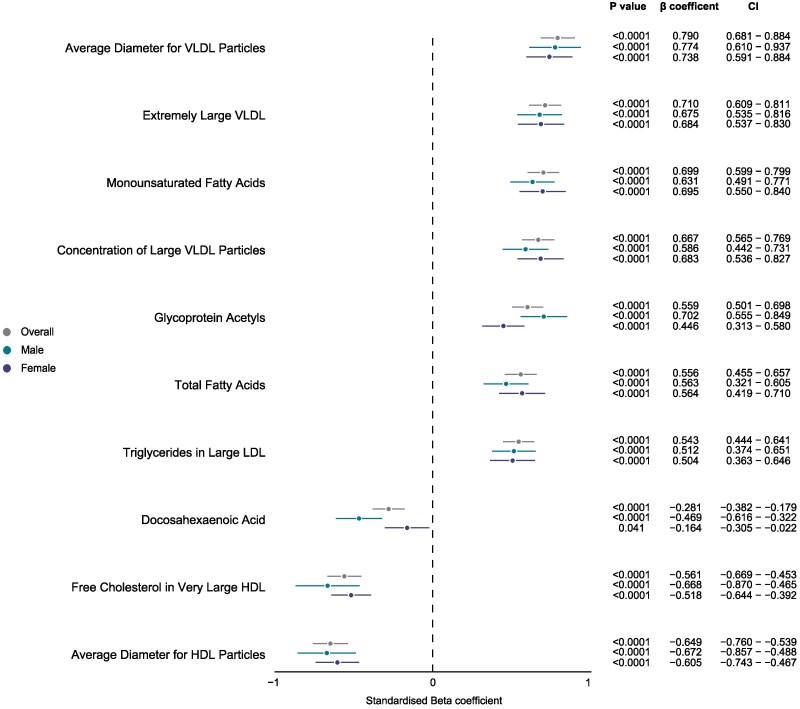
Associations between cardiovascular age-delta and NMR metabolites. Linear regression analysis of 10 significant metabolites with the largest effect sizes (seven positive and three negative) after Benjamini–Hochberg correction (*P* < 1 × 10^−4^) with cardiovascular age-delta (*n* = 22 534). Error bars indicate the beta coefficients' point estimates per standard deviation 95% confidence intervals, adjusted for age, age^2^, and statin users, and stratified by sex

We found positive associations between cardiovascular age-delta and apolipoprotein B, total cholesterol, and direct LDL cholesterol (all *P* < .0001), while HDL cholesterol was associated with a lower cardiovascular age-delta (*P* = .0071) as shown in *Figure 4B*. Using serum NMR metabolites, increased age-delta was linked to larger very low density lipoprotein (VLDL) particles, glycoprotein acetyls, and monosaturated fatty acid, while decreased age-delta was associated with HDL particle diameter, free cholesterol in very large HDL, and docosahexaenoic acid (*Figure 5*). Additionally, these biomarkers demonstrated changes across chronological age (see Supplementary data online, *Figure S5*).

E2 was associated with increased cardiovascular age-delta in males (*P* < .0001), but showed no significant association in all-age females (*P* = .0871) as shown in Supplementary data online, *Table S3* and *Figure 4C*. In pre-menopausal females only, E2 levels were linked to a modest decrease in cardiovascular age-delta (*P* = .0001).

An association between SHBG and increased age-delta was seen in females (*P* = .0437). Free testosterone was associated with a decrease in the cardiovascular age-delta in both sexes, as shown in *Figure 4C*.

### Physical activity and cardiovascular age-delta

We assessed whether physical activity modified the association between fat distribution and cardiovascular ageing. Fit-obese adults had a lower mean age-delta compared to unfit-obese adults (0.58 vs. 0.99, *P* = .007) (see Supplementary data online, *Table S8*). In fit-obese adults, visceral adipose tissue remained a significant positive predictor of age-delta [β = 0.194, (95% CI, .035 to .355), *P* = .017]. Taken with sex-stratified analyses of ageing by activity level and BMI group (see Supplementary data online, *Table S9*), this suggests that, while physical activity is associated with improved age-delta in obese adults, adverse fat distribution remains a consistent risk factor for accelerated ageing.

### Comparison of body mass index with fat mass

**Figure 6:**
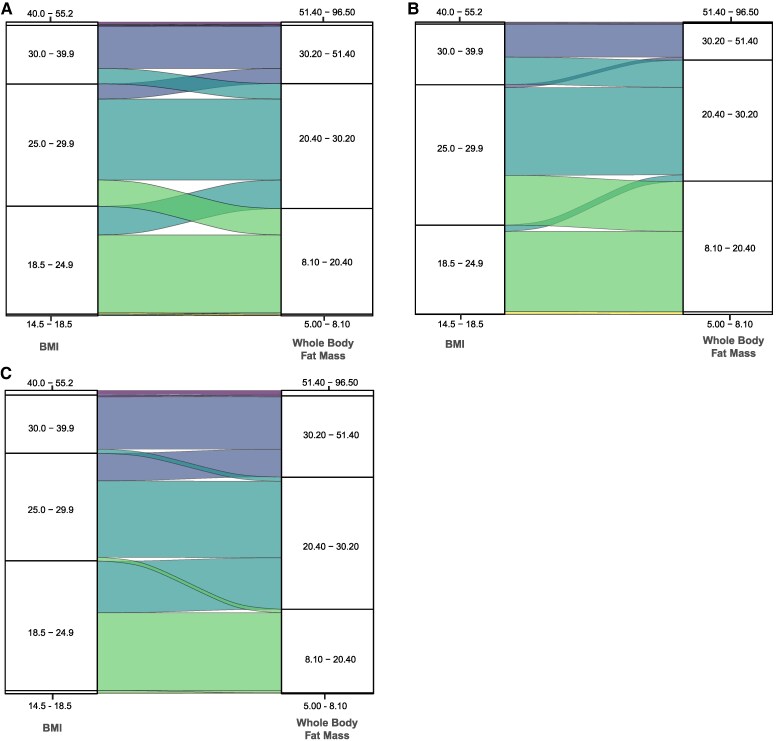
Reclassification of body mass index groups by whole-body fat mass. Series of alluvial plots that show the redistribution of participants in each body mass index (BMI) group to equivalent centile ranges of whole-body fat mass. *A*) Overall population (*n* = 21 241), *B*) females (*n* = 10 558), and *C*) males (*n* = 10 683)

Categorizing body composition by the same centile ranges as BMI-defined normal, overweight, and obese categories, we showed that 31% (*n* = 1141/3681) of overweight female participants fell into the ‘normal’ range for whole-body fat mass. Among overweight male participants, 11% (*n* = 537/4882) were reclassified to ‘normal’ whole-body fat mass, and 23% (*n* = 1123) into the ‘obese’ range (*Figure 6*).

### Cardiovascular outcome analysis

Incident atrial fibrillation [HR = 1.16, (95% CI, 1.02–1.31), *P* = .02] and type 2 diabetes [HR = 1.29, (95% CI, 1.08–1.54), *P* = .01] were associated with cardiovascular age-delta. The association between age-delta and MACE or all-cause mortality was not significant (see Supplementary data online, *Table S10*).

## Discussion

Obesity is a leading cause of cardiovascular disease independent of other risk factors,^34^ and in animal models causes a state of accelerated biological ageing of the heart and circulation through up-regulation of pro-fibrotic and inflammatory factors.^35,36^ Here, we show that fat distribution may underlie premature ageing of the human cardiovascular system and that phenotypic differences between sexes modify these processes. This work demonstrates the key role played by visceral adipose tissue and the distribution of subcutaneous fat in regulating biological age and highlights the potential value for novel therapies intended to extend health-span through modifying adipose tissue function.

Adipose tissue is a metabolically active distributed organ system composed of a variety of cell types that store energy and also contribute to the regulation of diverse biological functions through the secretion of cytokines, chemokines, and hormones.^37^ Adipose tissue is morphologically heterogeneous, with white, beige, and brown fat possessing increasing thermogenic adipocytes.^38^ Visceral and subcutaneous white adipose tissue depots are also developmentally distinct,^39^ with visceral fat associated with insulin resistance, local and systemic inflammation, and dyslipidemia.^40^ We showed that absolute visceral fat volume in women is 54% of that seen in men, while subcutaneous fat is 38% higher than in men. In middle-aged adults, while whole-body fat remains relatively stable with age, there is a progressive increase in muscle fat infiltration, a small rise in visceral fat volume, and a decline in subcutaneous fat in both sexes. Such age-related changes in adipose tissue are thought to involve a redistribution of fat depots and changes in their cellular composition, alongside a functional decline of adipocyte progenitors and accumulation of senescent cells.^41^ In animal models, widespread activation of immune cells is especially pronounced, with the earliest signs present in white adipose depots during middle age as part of an asynchronous pattern of inter-organ ageing.^42^

We used non-invasive imaging to provide an estimate of how an individual’s cardiovascular system has aged relative to a normative population, who were not obese or had major cardiovascular risk factors, and explore the association with fat phenotypes.^9^ We found that BMI was a weak predictor of age-delta in either sex, reflecting that accelerated ageing is not predicted by overall body mass. BMI also showed a sex bias in terms of over-representing women with normal fat mass as overweight and *vice versa* for men. These observations highlight the limitations and biases of aggregate measures such as BMI, and the potential for MRI-based body composition analysis, or accurate surrogate assessments, to personalize clinical risk prediction. Our data showed that visceral adipose tissue, liver fat, and to a lesser extent, muscle fat infiltration all predicted an increased age-delta in both sexes. Visceral adipose tissue promotes abnormal secretion of adipose-derived inflammatory cytokines and bioactive peptides, which are thought to promote premature brain ageing,^43,44^ and here, we show a potential shared mechanism with accelerated ageing of the heart and circulation that is independent of sex. We observed that glycoprotein acetyls, which are stable markers of cumulative inflammation over several years,^45^ were associated with accelerated ageing. We also found key sex differences with a gynoid fat distribution appearing protective for ageing in females and a potential causal association of genetically-determined gluteofemoral fat on attenuated cardiovascular ageing. Gluteofemoral fat is negatively correlated with cardiometabolic disease risk factors,^46^ and while the mechanism remains unclear, it may be partially mediated by the secretion of adiponectin, which enhances insulin sensitivity.^46^ However, gynoid fat was only associated with attenuated ageing in women, suggesting that hormonal factors that regulate fat distribution could also directly influence ageing.^47^ Oestrogens preferentially drive fat accumulation in the gluteofemoral depot rather than the visceral compartment and independently protect against atherosclerosis and endothelial dysfunction.^48,49^ Oestradiol plays a key role in the biology of ageing across organ systems,^11,50,51^ and our finding that it attenuates cardiovascular ageing in women until the menopause could be mediated through both fat-dependent and cardio-protective mechanisms. This supports a potential role for hormone replacement therapy in modulating cardiovascular ageing in women.

Our observation that visceral fat promotes ageing of the heart and circulation in humans provides support for the potential role of emerging treatments that target adipose tissue function to extend health-span. Glucagon-like peptide-1 receptor agonists (GLP-1 RAs), a class of antihyperglycemic drugs, are used to manage type 2 diabetes mellitus, but also have pleiotropic effects on protecting against age-related oxidative stress, cellular senescence, and chronic inflammation. They also substantially reduce visceral and liver fat in those with or without diabetes.^52^ Therefore, they may reduce both the volume of visceral fat as well as suppress secreted pro-inflammatory mediators of ageing.^53,54^ Human pharmacological interventions are considered as adjuncts to caloric restriction and physical activity, which may confer health benefits independently of weight loss. For age-associated diseases and lifespan, ERK, AMPK, and mTORC1 represent critical modifiable pathways. In animal models of ageing, IL11 is progressively up-regulated in liver, skeletal muscle, and fat to stimulate an ERK/AMPK/mTORC1 axis of cellular, tissue, and organismal ageing pathologies. Anti-IL11 therapy may reactivate age-repressed metabolic function in adipose tissue and is a potential therapeutic target for extending mammalian health-span.^55^ Adiposity-related chronic inflammation can respond to even mild changes in nutrient signalling in mouse models, suggesting a link between either dietary restriction or mTORC1 inhibition and ‘inflamm-ageing’.^56^ Together, this shows the potential for treatments that have pleiotropic effects on chronic sterile inflammation and apoptosis through mechanisms that include reprogramming of adipose tissue function.^57^ Emerging therapies, such as SGLT2 inhibitors and mitochondrial uncouplers, might also have a role in modulating the pro-ageing effects of adipose tissue function.

Our study has limitations. Older age groups and persons living in less socioeconomically deprived areas are under-represented in the UK Biobank.^58^ The population is predominantly European, and further work is required for people of diverse ancestries and social groups. For instance, people of Asian ancestry may have different sex-dependent distributions of fat compared to other ancestries and variation in gene-environment regulation of visceral adiposity,^59^ which may result in altered effects on cardiovascular ageing. This could also affect response to therapies directed at treating adverse fat phenotypes. Phenotyping is derived at a single time-point in this cross-sectional study, and we could not assess within-person trajectories nor fully account for differential cohort and periodic effects. We were not able to externally validate the findings as there is no other biobank study that collects equivalent phenotypic data at the required scale; however, cardiovascular age-delta satisfies several proposed evaluation criteria as a useful biomarker of ageing.^24^ Future work will seek to generalize these findings through approaches that can be used on more widely available phenotypic data, in particular to study the effects of ancestral diversity. Further research using longitudinal data would help to understand how biological ageing mediates the effect of risk exposures on outcomes in the cardiovascular system and repeat imaging in the UK Biobank will help to address this. While we observe an association with early risk outcomes, the effects of age-related damage accumulation on major events and mortality may only become apparent in geriatric populations.^24^ Atherosclerosis is associated with premature biological ageing, and although we could not assess coronary or peripheral plaque with CMR-derived phenotypes, we were able to quantify downstream effects on correlated changes to vascular distensibility.^60^ While we assessed the effects of physical activity, direct measurement of VO_2_max would have provided quantified data on exercise capacity. Diet and medications also remain to be explored as potential modifiers. The two samples used for causal inference were drawn from different studies in the UK Biobank, but with partial overlap of participants, which may increase the potential for over-fitting and weak instrument bias. Analysis of independent samples would strengthen the genetic evidence for causality. In general, genome wide association studies (GWAS) prioritize pleiotropic genes, particularly for complex traits, which may introduce false positive MR causal estimates for the type of relationships we study here.^61^

In conclusion, sex-dependent fat phenotypes are related to biological cardiovascular ageing in a predominantly middle-aged white population, which highlights adipose tissue distribution and function as potential targets for interventions to extend healthy lifespan and to support public health strategies.^62^

## Supplementary Material

ehaf553_Supplementary_Data

## Declarations

### Data Availability

The scripts for data analysis are publicly available https://github.com/ImperialCollegeLondon/adiposity_aging. Data from the UK Biobank are available for approved research.
