# Supplementary material for: Sex-specific body fat distribution predicts cardiovascular ageing
Source: Eur Heart J. 2025 Aug 22;46(46):5076–88. doi: 10.1093/eurheartj/ehaf553 (PMC12682380; doi:10.1093/eurheartj/ehaf553)
Supplement: ehaf553_Supplementary_Data [file ehaf553_supplementary_data.pdf]

Supplemental Material

Supplemental Methods

Statistical modelling and data standardisation for fat phenotypes

For each fat phenotype and circulating biomarker, we fitted three linear models: one for males, one for females, and one for both sexes combined.

$$\begin{aligned}\text{Dependent Variable} = \beta_0 + \beta_1 \times \text{Independent Variable 1}\\ + \beta_2 \times \text{Independent Variable 2}\\ + \beta_3 \times \text{Independent Variable 3}\\ + \beta_4 \times \text{Independent Variable 4}\\ + \varepsilon\end{aligned}$$

Additionally, to account for potential effects of body size on fat distribution, we adjusted for height-squared to control for variation in stature, following the principles of allometric scaling.[29]

Given that the extracted data included various measurements for adipose tissue, we standardised all available data to ensure accurate statistical calculations. Raw data for visceral adipose tissue (VAT), abdominal subcutaneous adipose tissue (ASAT), muscle adipose tissue infiltration (MATI), liver proton density fat fraction (PDFF), total abdominal adipose tissue (TAAT), android and gynoid adipose tissue mass, total trunk fat mass (TTFM), whole body fat mass (WBFM), apolipoprotein A and B, direct low-density lipoprotein, high-density lipoprotein, triglycerides, and cholesterol underwent Box-Cox normalisation to meet the assumption of normally distributed residuals.

$$y^{(\lambda)} = \begin{cases} \frac{y^{\lambda}-1}{\lambda} & \text{if } \lambda \neq 0, \\ \ln(y) & \text{if } \lambda = 0. \end{cases}$$

When the value of a predictor variable increases by one standard deviation, the outcome variable is expected to increase by the amount of the coefficient associated with that predictor in the regression model. This coefficient indicates the change in the outcome variable for each one standard deviation increase in the predictor variable, while keeping all other variables constant.

$$\beta_{\text{std}} = (\text{SD}_X)\beta/\text{SD}_Y$$

The 95% confidence interval (CI) for the effect estimates was calculated using the formula:

$$\beta \pm 1.96 \times \text{SE}(\beta)$$

The Pearson correlation coefficient was used to examine the linear relationships between clinical variables, such as adipose tissue phenotypes, circulating biomarkers, and their effect on age-delta.

$$r = \frac{\sum(X_i - \overline{X})(Y_i - \overline{Y})}{\sqrt{\sum(X_i - \overline{X})^2 \sum(Y_i - \overline{Y})^2}}$$

Quantification of cardiovascular age-delta

We used a pre-trained model developed to predict cardiovascular age using image-derived phenotypes from cardiac MRI, based on 39,559 UK Biobank participants.[9] It was initially trained on 5,065 healthy individuals, free from cardiac, metabolic, or respiratory diseases, with a body mass index below 30. These participants were divided into training (80%, n = 4,019) and test (20%, n = 1,044) sets. CatBoost, a gradient boosting algorithm, was used with default hyperparameters and early stopping rounds set between 50 and 100. Hyperparameter tuning was performed using a 10% validation holdout (n = 403) (using Python package `Optuna`). A further 10% (n = 362) was used for internal early stopping, leaving 3,256 instances for final training. Thirty models with different random seeds were trained, and the one with the lowest mean absolute error on the holdout set was selected. This model predicted age in the remaining 34,147 participants. To correct for bias between predicted age-delta and chronological age, a linear regression was conducted. The regression slope and intercept were used to calculate an offset, which was subtracted from the uncorrected predicted age, yielding the corrected cardiovascular age.

The machine learning model trained on healthy participants (n=4019), applied on a holdout test set (n=1044), had a coefficient of determination (R^2) of 0.49, a Pearson correlation coefficient ($|r|$) between predicted age and chronological age of 0.70 and a mean absolute error (MAE) of 4.2 years.

Quantile-based categorisation of BMI and corresponding whole body fat mass

To categorise whole body fat mass (WBFM) based on specific Body Mass Index (BMI) quantiles, a systematic approach involving the calculation of empirical cumulative distribution functions (ECDF) for both BMI and WBFM distributions was utilised (Supplementary Figure 1). The detailed steps are as follows:

Dataset preparation: The dataset containing BMI and WBFM measurements was loaded and prepared for analysis. Specifically, key quantiles within the BMI distribution were identified and mapped onto the WBFM distribution.

Empirical cumulative distribution function (ECDF): For a given dataset $X = \{x_1, x_2, \dots, x_n\}$, the ECDF is defined as:

$$\hat{F}_n(x) = \frac{1}{n} \sum_{i=1}^{n} \mathbb{1}(x_i \leq x)$$

where $\mathbb{1}(x_i \leq x)$ is the indicator function, which is 1 if $x_i \leq x$ and 0 otherwise. This function provides a stepwise cumulative probability for each value in the dataset.

Quantile calculation for BMI: The ranks of the BMI values were calculated, and the ECDF was applied to determine the quantile for each specified BMI value. Specifically, for a BMI value b, the quantile Q_b is:

$$Q_b = \min\left(\hat{F}_n(b) \mid b_i \geq b\right)$$

This quantile Q_b indicates the proportion of the dataset with BMI values less than or equal to b.

Mapping BMI quantiles to WBFM: Using the BMI quantiles obtained, the corresponding WBFM values at these quantiles were identified. For a quantile Q_b, the WBFM value w_b is calculated as:

$$w_b = \text{Quantile}(\text{WBFM}, Q_b)$$

where $\text{Quantile}(\text{WBFM}, Q_b)$ is the WBFM value at the specified quantile Q_b.

Visualisation: The distributions of BMI and WBFM were visualised with specified quantile lines. Histograms were plotted for both distributions, and vertical lines indicating the specified BMI quantiles and corresponding WBFM values were superimposed. This dual-distribution plot provides a clear visual representation of the relationship between BMI and WBFM across the quantiles.

Outcome analysis

Cox proportional hazards regression was used to examine the relationships between cardiovascular age-delta, adjusted for age and sex, and all cause mortality, composite (MACE) and individual cardiovascular outcomes, including hypertension, atrial fibrillation, stroke, angina, heart failure, myocardial infarction (MI), and type 2 diabetes. Cardiovascular age-delta was analyzed as a continuous variable (mean-centered). The model demonstrated statistical significance ($\chi^2 = 30.68, P < 0.001$) with modest discriminatory ability (concordance index: 0.55).

$$h(t \mid X) = h_0(t)\exp\left(\beta_1(\text{ca\_delta\_mean\_centered}) + \beta_2(\text{age}) + \beta_3(\text{sex}) + \sum_{i=4}^{n} \beta_i(\text{outcome}_i)\right)$$

ICD Methodology for Outcome Ascertainment

All cardiovascular events and related outcomes were identified and confirmed using International Classification of Diseases (ICD) codes in electronic health records. Supplementary table 1 lists the primary ICD-9 and ICD-10 codes used to define each outcome. Where multiple codes overlapped for a single disease category, we included all relevant codes to maximize case capture. Discrepancies or borderline cases were reviewed by clinical adjudicators when possible.

Composite Cardiovascular Events

A composite outcome (major adverse cardiovascular events, MACE) was defined as any hospitalization or clinical encounter with a diagnosis of *(for example, MI, stroke, or heart failure)* using the codes above. Events were enumerated from the date of each participant's CMR visit until the end of follow-up or the first qualifying event.

Fitness-BMI Categorization and Statistical Analysis

Fitness was assessed using MET-based physical activity scores, with participants categorized into fitness-fatness groups based on MET thresholds ($\geq$ 1200 MET-minutes/week as "Fit" and $<$ 1200 MET-minutes/week as "Unfit") and BMI classifications ("Normal Weight," "Overweight," "Obese"). These categories were combined (e.g., "Fit-Normal Weight," "Unfit-Overweight") to evaluate their impact on cardiovascular age-delta. Pairwise comparisons between fitness-fatness groups were conducted using the Tukey HSD (Honestly Significant Difference) test to identify statistically significant differences while controlling for Type I error rates. Analyses were further stratified by sex.

Tukey HSD Formula

The Tukey HSD test calculates pairwise differences between group means and uses the following formula for the test statistic:

$$q = \frac{|\bar{X}_i - \bar{X}_j|}{SE}$$

where:

- $\bar{X}_i$ and $\bar{X}_j$ are the means of the two groups being compared,
- SE is the standard error of the mean differences, calculated as:

$$SE = \sqrt{\frac{MSE}{n}}$$

- MSE is the mean squared error from the ANOVA,
- n is the sample size per group (assumed equal for simplicity).

The critical value for q is determined based on the Tukey distribution for the number of groups and degrees of freedom from the ANOVA.

Statistical significance is achieved when the observed q value exceeds the critical value at the specified α level (e.g., $\alpha = 0.05$).

Missing Data Handling

We merged data from all relevant sources (including image-derived phenotypes, questionnaire responses, and clinical measurements) using a unique participant identifier in Python's pandas library. After aligning variable names and checking for consistency across datasets, we identified rows containing missing values in any of the primary variables needed for our analyses (e.g., fat phenotypes, therapy indicators, or covariates). We then applied complete-case analysis by dropping those rows via DataFrame.dropna() to retain only participants with no missing values in the key variables of interest. This step ensures that all variables used in our regression models have the same valid observations. While complete-case analysis can reduce sample size, it avoids assumptions required for imputation of complex measures (e.g., image-derived traits) and maintains a consistent dataset across all variables examined. The proportion of missing data was relatively small, and we determined that omitting those cases would not substantially bias our final results.

For hormone measurements and android/gynoid fat mass we created separate datasets. Because these variables were only available for a limited number of participants, combining them with the larger cohort would further reduce sample size and potentially bias the primary analyses. This workflow was documented in Python to ensure reproducibility and transparency of the final analytic samples.

Supplementary Tables

Supplementary Table 1. ICD-9 and ICD-10 Codes Used to Define Outcomes

Outcome	ICD-9 Codes	ICD-10 Codes
**Hypertension**	401.0, 401.1, 401.9, 402.00, 402.01, 402.10, 402.11, 402.90, 402.91, 403.00, 403.01, 403.10, 403.11, 403.90, 403.91, 404.00, 404.01, 404.10, 404.11, 404.90, 404.91, 405.01, 405.09, 405.11, 405.19, 405.91, 405.99	I10, I11.0, I11.9, I12.0, I12.9, I13.0, I13.10, I13.11, I13.2, I15.0, I15.1, I15.2, I15.8, I15.9
**Atrial Fibrillation**	427.31	I48.0, I48.1, I48.2, I48.91
**Stroke**	430, 431, 432.0, 432.1, 432.9, 433.00, 433.01, 433.10, 433.11, 433.20, 433.21, 433.30, 433.31, 433.80, 433.81, 433.90, 433.91, 434.00, 434.01, 434.10, 434.11, 434.90, 434.91, 436	I60.0–I60.9, I61.0–I61.9, I62.00–I62.9, I63.00–I63.9, I64
**Angina**	413.0, 413.1, 413.9	I20.0, I20.1, I20.8, I20.9
**Heart Failure**	428.0, 428.1, 428.20, 428.21, 428.22, 428.23, 428.30, 428.31, 428.32, 428.33, 428.40, 428.41, 428.42, 428.43, 428.9	I50.1, I50.20, I50.21, I50.22, I50.23, I50.30, I50.31, I50.32, I50.33, I50.40, I50.41, I50.42, I50.43, I50.9
**Myocardial Infarction**	410.00–410.92, 412	I21.01–I21.4, I22.0–I22.9, I25.2
**Type 2 Diabetes**	250.00–250.93	E11.0–E11.9

Supplementary Table 2. Summary of fat phenotypes by sex and ancestry. VAT, visceral adipose tissue; ASAT, abdominal subcutaneous adipose tissue; TTFM, total trunk fat mass; WBFM, whole body fat mass; TAAT, total abdominal adipose tissue; MATI, muscle adipose tissue infiltration. IQR, interquartile range.

Phenotype	Sex	Ancestry	1st Quartile (0.25)	Median (0.5)	3rd Quartile (0.75)	IQR
VAT	Female	White background	1.58	2.47	3.65	2.07
VAT	Female	Black background	1.50	2.33	2.92	1.42
VAT	Female	Asian background	1.69	2.83	3.95	2.26
VAT	Female	Mixed background	1.52	2.57	3.83	2.31
VAT	Male	White background	3.34	4.83	6.47	3.13
VAT	Male	Black background	3.18	4.67	6.45	3.26
VAT	Male	Asian background	3.69	4.82	6.52	2.83
VAT	Male	Mixed background	3.18	5.00	6.55	3.36
ASAT	Female	White background	5.82	7.75	10.15	4.33
ASAT	Female	Black background	6.12	8.11	9.93	3.80
ASAT	Female	Asian background	5.96	8.16	11.11	5.15
ASAT	Female	Mixed background	6.17	8.03	9.87	3.70
ASAT	Male	White background	4.29	5.50	7.07	2.78
ASAT	Male	Black background	4.42	5.91	6.73	2.31
ASAT	Male	Asian background	4.69	5.84	7.11	2.41
ASAT	Male	Mixed background	4.07	5.15	6.59	2.52
TTFM	Female	White background	9.90	12.80	16.20	6.30
TTFM	Female	Black background	10.70	13.20	17.00	6.30
TTFM	Female	Asian background	10.25	13.00	17.85	7.60
TTFM	Female	Mixed background	9.85	12.40	16.45	6.60
TTFM	Male	White background	10.80	13.50	16.60	5.80
TTFM	Male	Black background	10.97	13.70	17.25	6.28
TTFM	Male	Asian background	11.70	14.20	16.65	4.95
TTFM	Male	Mixed background	10.75	12.70	16.05	5.30
WBFM	Female	White background	19.80	25.00	31.40	11.60
WBFM	Female	Black background	21.30	26.30	32.00	10.70
WBFM	Female	Asian background	20.18	25.95	33.90	13.72
WBFM	Female	Mixed background	20.30	24.45	32.12	11.82
WBFM	Male	White background	17.00	21.10	26.30	9.30
WBFM	Male	Black background	17.43	21.65	27.23	9.80
WBFM	Male	Asian background	18.65	22.20	26.75	8.10
WBFM	Male	Mixed background	16.82	20.55	25.43	8.60
TAAT	Female	White background	0.49	0.56	0.62	0.14
TAAT	Female	Black background	0.48	0.55	0.61	0.12
TAAT	Female	Asian background	0.48	0.57	0.65	0.17
TAAT	Female	Mixed background	0.50	0.56	0.63	0.13
TAAT	Male	White background	0.40	0.46	0.52	0.13
TAAT	Male	Black background	0.39	0.45	0.52	0.13
TAAT	Male	Asian background	0.41	0.46	0.52	0.11
TAAT	Male	Mixed background	0.37	0.45	0.52	0.15
MATI	Female	White background	6.63	7.66	8.91	2.28
MATI	Female	Black background	6.58	7.67	8.61	2.03
MATI	Female	Asian background	6.77	7.91	9.22	2.45
MATI	Female	Mixed background	6.88	7.96	8.93	2.05
MATI	Male	White background	5.66	6.58	7.72	2.06
MATI	Male	Black background	5.53	6.84	7.58	2.05
MATI	Male	Asian background	5.67	6.73	7.76	2.09
MATI	Male	Mixed background	5.59	6.44	7.45	1.86

Supplementary Table 3. Association of fat phenotypes, cardiometabolic and endocrine biomarkers with cardiovascular age-delta. Sex stratified β coefficients, 95% confidence intervals and *P* values for each predictor with age-delta as the dependent variable in a linear regression model with age and age^2 as covariates.

Trait		*β* coefficient	95% CI	*P* value
**Body fat phenotypes**				
Visceral adipose tissue (VAT)	Overall	0.656	0.537 - 0.775	< 0.0001
	Female	0.497	0.323 - 0.671	0.0007
	Male	0.739	0.577 - 0.9	< 0.0001
Abdominal subcutaneous adipose tissue (ASAT)	Overall	0.154	0.045 - 0.264	0.0069
	Female	-0.099	-0.246 - 0.047	0.1981
	Male	0.432	0.269 - 0.596	< 0.0001
Muscle adipose tissue infiltration (MATI)	Overall	0.183	0.112 - 0.224	0.0003
	Female	0.482	0.321 - 0.692	< 0.0001
	Male	0.283	0.198 - 0.376	0.0007
Liver proton density fat fraction (PDFF)	Overall	1.066	0.835 - 1.298	< 0.0001
	Female	0.991	0.675 - 1.306	< 0.0001
	Male	1.13	0.788 - 1.472	< 0.0001
Total abdominal adipose tissue (TAAT)	Overall	0.615	0.499 - 0.732	< 0.0001
	Female	0.355	0.205 - 0.505	0.0007
	Male	0.949	0.767 - 1.132	< 0.0001
Android adipose tissue mass	Overall	0.527	0.292 - 0.761	0.0002
	Female	0.121	-0.199 - 0.441	0.4727
	Male	0.983	0.64 - 1.326	< 0.0001
Gynoid adipose tissue mass	Overall	0.077	-0.175 - 0.329	0.5479
	Female	-0.499	-0.85 - -0.149	0.0003
	Female aged (Premenopause)	- 0.198	-0.084 - -0.027	0.0011
	Female aged (Postmenopause)	0.101	-0.020 - 0.220	0.441
	Male	0.688	0.33 - 1.046	0.0066
**Body fat composition**				
Total trunk fat mass	Overall	-0.15	-0.165 - 0.41	0.0408
	Female	-0.403	-0.821 - -0.14	0.0061
	Female aged (Premenopause)	- 0.0399	-0.0062 - -0.0018	0.031
	Female aged (Postmenopause)	0.051	-0.00103 - 0.00904	0.048
	Male	0.415	0.032 - 0.211	0.0343
Whole body fat mass	Overall	0.011	-0.12 - 0.02	0.0431
	Female	-0.389	-0.732 - -0.254	0.0043
	Male	0.428	0.071 - 0.114	0.0191
**Body mass index**	Overall	-0.025	-0.048 - -0.001	0.0430
	Female	-0.85	-0.115 - -0.055	0.0092
	Male	0.063	0.026 - 0.1	0.0813
**Cardiometabolic biomarkers**				
Apolipoprotein A	Overall	-0.003	-0.125 - 0.12	0.9673
	Female	-0.12	-0.293 - 0.053	0.1946
	Male	0.09	-0.084 - 0.257	0.3413
Apolipoprotein B	Overall	0.336	0.224 - 0.448	< 0.0001
	Female	0.209	0.052 - 0.33	0.0117
	Male	0.214	0.058 - 0.37	0.0117
Direct low-density Lipoprotein	Overall	0.287	0.176 - 0.399	<0.0001
	Female	0.15	0.029 - 0.388	0.0423
	Male	0.18	0.024 - 0.336	0.0310
High-density lipoprotein cholesterol	Overall	-0.187	- 0.311 - -0.063	0.0071
	Female	-0.331	-0.535 - -0.1	0.0053
	Male	-0.261	-0.435 - -0.088	0.0072
Triglycerides	Overall	0.569	0.459 - 0.679	0.0062
	Female	0.652	0.485 - 0.858	0.0073
	Male	0.671	0.485 - 0.858	0.0072
Cholesterol (total)	Overall	0.304	0.191 - 0.417	< 0.0001
	Female	0.183	0.048 - 0.373	0.0219
	Male	0.197	0.04 - 0.353	0.0207
**Endocrine biomarkers**				
Sex hormone binding globulin (SHBG)	Overall	0.107	-0.356 - 0.57	0.6497
	Female	0.36	-0.245 -0.965	0.0437
	Male	-0.362	-1.068 - 0.344	0.3546
Testosterone (free form)	Overall	-0.063	-0.082 - -0.043	< 0.0001
	Female	-0.04	-0.066 - -0.014	0.0071
	Male	-0.0811	-0.109 - -0.052	< 0.0001
Oestradiol (E2)	Overall	0.189	-0.259 - 0.679	0.0862
	Female	- 0.061	-0.685 - 0.558	0.0871
	Female (Premenopause)	- 0.00499	-0.000772 - -0.000226	0.0001
	Female (Postmenopause)	0.000301	-0.000105 - 0.000707	0.1424
	Male	0.481	0.245 - 0.717	< 0.0001

Supplementary Table 4. Sex-specific effects of fat phenotypes on age-delta. Beta(Fem) is the estimated increase in age-delta per unit of the phenotype for females, Beta(Male) is the estimated increase for males, SE is the standard error of the estimate, t is the t-statistic, p is the p-value, and Diff(M-F) is the difference between male and female slopes.

Phenotype	Beta(Fem)	SE(Fem)	t(Fem)	p(Fem)	Beta(Male)	SE(Male)	t(Male)	p(Male)	Diff(M-F)	SE(Diff)	t(Diff)	p(Diff)
Visceral adipose tissue (VAT)	0.272000	0.050000	5.440000	<0.0001	0.360000	0.035000	10.285714	<0.0001	0.088000	0.015000	5.866667	<0.0001
Abdominal subcutaneous adipose tissue (ASAT)	-0.064698	0.021813	-2.966049	0.0030	0.069661	0.027320	2.549840	0.0108	0.134360	0.034913	3.848464	0.0001
Total trunk fat mass (TTFM)	-0.089749	0.015413	-5.823045	<0.0001	0.058757	0.015542	3.780451	0.0002	0.148506	0.021905	6.779633	<0.0001
Whole body fat mass (WBFM)	-0.049034	0.008210	-5.972487	<0.0001	0.027119	0.009155	2.962109	0.0031	0.076153	0.012295	6.193891	<0.0001
Muscle adipose tissue infiltration (MATI)	0.106157	0.031955	3.322039	0.0009	0.145347	0.034810	4.175381	<0.0001	0.039189	0.046783	0.837681	0.4022
Android fat mass	-0.000117	0.000103	-1.140544	0.2541	0.000562	0.000132	4.255650	<0.0001	0.000680	0.000167	4.059421	<0.0001
Gynoid fat mass	-0.000148	0.000062	-2.387000	0.0170	0.000447	0.000124	3.601237	0.0003	0.000572	0.000155	3.686862	0.0002

Supplementary Table 5. Multivariable associations of age-delta with adipose tissue phenotypes, diabetes, and antidiabetic therapies.
Four therapy categories: **diabetes**, **Biguanides (Metformin)**, **Sulfonylurea** (glimepiride, gliclazide, glipizide), **Thiazolidinediones (pioglitazone, rosiglitazone)**, and **Insulin**.
There are 1833 patients with diabetes in total: Biguanides (n=1493), Sulfonylurea (n=148), Thiazolidinediones (n=49), Insulin (n=141).
Abbreviations: MATI = muscle adipose tissue infiltration, ASAT = abdominal subcutaneous adipose tissue, VAT = visceral adipose tissue.

Variable	coef	std err	t	P>|t|	CI
Intercept	-3.1904	0.485	-6.577	0.0003	(-4.142, -2.239)
Sex	0.1443	0.126	1.148	0.251	(-0.103, 0.392)
Diabetes	3.4281	1.420	2.414	0.016	(0.646, 6.210)
VAT : Diabetes	2.8810	1.350	2.135	0.033	(0.230, 5.532)
ASAT : Diabetes	2.7725	1.530	1.812	0.070	(-0.227, 5.772)
MATI : Diabetes	3.1010	1.200	2.584	0.010	(0.750, 5.452)
VAT: Diabetes: TherapyGroup[Biguanides (Metformin)]	0.4812	0.210	2.293	0.022	(0.070, 0.893)
ASAT: Diabetes: TherapyGroup[Biguanides (Metformin)]	0.1989	0.045	4.420	0.0001	(0.111, 0.287)
MATI: Diabetes: TherapyGroup[Biguanides (Metformin)]	0.1231	0.029	4.243	0.0001	(0.066, 0.180)
VAT: Diabetes: TherapyGroup[Sulfonylureas (Glimepiride, Gliclazide, Glipizide)]	-0.1581	0.274	-0.577	0.564	(-0.695, 0.379)
ASAT: Diabetes: TherapyGroup[Sulfonylureas (Glimepiride, Gliclazide, Glipizide)]	0.1210	0.058	2.086	0.037	(0.008, 0.234)
MATI: Diabetes: TherapyGroup[Sulfonylureas (Glimepiride, Gliclazide, Glipizide)]	0.0290	0.027	1.074	0.283	(-0.024, 0.082)
VAT: Diabetes: TherapyGroup[Thiazolidinediones (Pioglitazone, Rosiglitazone)]	-0.1342	0.210	-0.639	0.523	(-0.545, 0.277)
ASAT: Diabetes: TherapyGroup[Thiazolidinediones (Pioglitazone, Rosiglitazonee)]	0.0765	0.046	1.663	0.096	(-0.014, 0.167)
MATI: Diabetes: TherapyGroup[Thiazolidinediones (Pioglitazone, Rosiglitazone)]	-0.0331	0.025	-1.324	0.186	(-0.081, 0.015)
VAT: Diabetes: TherapyGroup[Insulin]	0.0150	0.022	0.666	0.506	(-0.027, 0.057)
ASAT: Diabetes: TherapyGroup[Insulin]	0.0280	0.012	2.333	0.020	(0.004, 0.052)
MATI: Diabetes: TherapyGroup[Insulin]	0.0190	0.017	1.118	0.264	(-0.014, 0.052)

Supplementary Table 6. Genetic instruments for Mendelian randomisation. Gluteofemoral adipose tissue (GFAT), visceral adipose tissue (VAT), abdominal subcutaneous adipose tissue (ASAT) genetic variants Used in MR analysis on causal association to the cardiovascular age (outcome). Columns include the dbSNP ID, GRCh37 position, effect allele and the effect allele frequency (EAF), and the Genome-Wide Association Study (GWAS) stats for each Single Nucleotide Polymorphism (SNP) on exposure and outcome (Beta, SE, *P* value).

							Exposure			Outcome		
	**SNP**	**CHROM**	**POS**	Effect Allele	Other Allele	EAF (Exposure)	Beta	SE	*P* value	Beta	SE	*P* value
Exposure: GFAT (ID: gfatadjbmi3)												
1	rs10044492	5	167634	C	T	0.732286	-0.0478462	0.00805882	5.30E-09	0.0371827	0.0714443	0.602758
2	rs10501153	11	563572	C	T	0.677439	-0.0439302	0.0076186	5.90E-09	-0.071416	0.0675313	0.290281
3	rs11205303	1	165637	T	C	0.596291	-0.0392991	0.00724463	1.70E-08	0.126362	0.064481	0.0500429
4	rs114078082	6	544713	G	A	0.958096	0.138123	0.0177313	2.00E-15	-0.126356	0.154574	0.41368
5	rs12814794	12	460442	G	A	0.247599	-0.0721238	0.00826435	1.60E-18	-0.118934	0.073571	0.105977
6	rs13099700	3	226439	A	G	0.721677	0.0471756	0.00794479	7.90E-09	-0.055496	0.0705424	0.431461
7	rs13142096	4	730465	A	G	0.727366	-0.0470762	0.00801245	8.40E-09	0.160797	0.0712461	0.0240202
8	rs13389219	2	18149	C	T	0.606906	-0.0731577	0.00725328	3.00E-23	0.0865677	0.0644427	0.179176
9	rs1469246	4	166086	G	A	0.671272	-0.0540125	0.0075806	9.40E-13	0.104254	0.0673262	0.121513
10	rs1907218	10	152883	T	C	0.314461	-0.0488068	0.00764797	3.60E-10	0.00286916	0.0677417	0.966216
11	rs2082162	5	598391	G	T	0.412166	-0.038786	0.00725607	2.20E-08	-0.0525403	0.0643704	0.414382
12	rs2267373	22	471412	C	T	0.418883	0.0461893	0.00722912	1.40E-10	-0.00671632	0.0641773	0.916652
13	rs2300669	3	620155	C	A	0.615258	-0.0421979	0.00727957	4.40E-09	-0.0163743	0.0645603	0.799784
14	rs2943653	2	24424	C	T	0.325957	0.0751004	0.0075487	6.90E-23	0.0105112	0.0671747	0.875659
15	rs2955617	17	196865	C	A	0.348318	-0.0418669	0.00747226	1.20E-08	0.0837415	0.066523	0.2081
16	rs3822072	4	104928	G	A	0.545788	0.0478524	0.00712951	4.90E-12	-0.145465	0.0631734	0.021307
17	rs3936511	5	681508	A	G	0.808734	0.0815883	0.00901579	3.90E-20	-0.0777822	0.0805452	0.334204
18	rs4450871	4	879764	A	G	0.554794	-0.0383337	0.0071357	3.10E-08	0.0306861	0.0632487	0.627562
19	rs4759309	12	682774	G	A	0.221429	-0.0443202	0.00853853	4.20E-08	-0.00480906	0.0763794	0.949797
20	rs546560809	4	134628	T	G	0.961183	0.0981811	0.0184826	2.50E-08	-0.237203	0.1644	0.149074
21	rs71304101	3	297793	G	A	0.879021	-0.0618006	0.0108921	1.70E-09	-0.0166699	0.0969471	0.86348
22	rs7133378	12	14789	G	A	0.679988	-0.0876372	0.00761231	5.60E-29	0.245266	0.0674336	0.000276149
23	rs72959041	6	132816	G	A	0.952589	0.195251	0.0168711	3.20E-32	0.0319367	0.148534	0.829759
24	rs8075019	17	444844	G	A	0.872483	0.0634833	0.0107754	2.30E-10	-0.0383858	0.0965028	0.690803
25	rs998584	6	677005	C	A	0.516864	0.07952	0.00711591	6.10E-31	-0.188181	0.0631683	0.00289384
Exposure: VAT (ID: vatadjbmi3)												
1	rs11031796	11	498806	G	A	0.611683	0.0524669	0.00728182	5.10E-14	-0.0451491	0.0646797	0.485157
2	rs11992444	8	565812	G	T	0.491592	-0.0779981	0.00709912	1.30E-29	-0.10997	0.0632918	0.0823087
3	rs12089366	1	225654	C	T	0.776758	0.0580131	0.00855551	9.40E-12	-0.0365977	0.0765896	0.632767
4	rs1329254	10	50472	C	T	0.369978	0.0418348	0.00733732	1.40E-08	-0.081558	0.0651416	0.210576
5	rs1635851	7	463016	C	T	0.414446	0.040899	0.00720247	3.80E-08	-0.0305015	0.064236	0.634908
6	rs30351	5	680081	G	A	0.26445	0.0705595	0.00808072	1.10E-16	-0.033718	0.0724539	0.641669
7	rs35932591	2	142074	C	T	0.878816	0.0606447	0.0107886	3.80E-08	0.0379672	0.0967951	0.694882
8	rs3731861	2	233585	T	C	0.62196	-0.0382281	0.00730702	4.70E-08	0.0661645	0.0654018	0.311708
9	rs4307676	11	66432	G	A	0.833929	0.0535154	0.00949868	8.80E-09	0.104528	0.0840798	0.213805
10	rs4872393	8	575554	G	A	0.7734	-0.0601652	0.00844375	2.00E-12	0.286302	0.0758622	0.00016098
11	rs56006999	1	224281	C	T	0.821332	0.0536419	0.00922851	3.60E-09	0.0485811	0.0830658	0.558653
12	rs56082403	3	171233	T	C	0.593073	-0.0556812	0.00723565	6.90E-14	0.0200518	0.0647187	0.756692
13	rs577721086	6	132815	T	C	0.952316	-0.117892	0.01673	5.20E-13	0.060537	0.147915	0.682346
14	rs7133378	12	14789	G	A	0.679988	0.0458609	0.00758948	6.60E-10	0.245266	0.0674336	0.000276149
15	rs72810972	5	192967	G	T	0.716377	-0.0539032	0.00784907	2.30E-12	-0.0534721	0.0694006	0.441019
16	rs7933253	11	762768	T	C	0.0482717	0.0978848	0.0167983	1.30E-08	0.249213	0.152765	0.102826
17	rs998584	6	677005	C	A	0.516864	-0.0570321	0.00708399	1.80E-15	-0.188181	0.0631683	0.00289384
Exposure: ASAT (ID: asatadjbmi3)												
1	rs1159619	6	132699	C	A	0.544561	0.0457682	0.0071716	1.20E-10	0.0367581	0.0630816	0.560095
2	rs11709077	3	297621	G	A	0.88011	-0.0696508	0.0110061	1.70E-10	-0.00247357	0.0973027	0.979719
3	rs13322435	3	171233	A	G	0.59074	0.0570714	0.00731172	2.40E-15	0.028349	0.0647402	0.66147
4	rs17205757	15	860493	A	G	0.673992	-0.0415864	0.00765851	3.20E-08	0.00483059	0.0676445	0.943071
5	rs1779445	1	150977	T	C	0.194296	-0.0493135	0.00906223	1.90E-08	0.101266	0.0806485	0.209254
6	rs1815172	15	108655	C	T	0.476133	-0.0594399	0.00716879	6.80E-17	-0.0705533	0.0630489	0.263139
7	rs2302209	19	448214	C	T	0.7193	-0.0461843	0.00799643	3.40E-09	-0.114164	0.0703054	0.104423
8	rs2943647	2	244257	T	C	0.348169	0.0432106	0.00746638	5.80E-09	0.016165	0.0658683	0.806138
9	rs3850625	1	217777	G	A	0.88219	-0.0786112	0.0110023	1.80E-12	-0.0327814	0.0976972	0.737219
10	rs3936510	5	681509	G	T	0.798132	-0.0629779	0.00887846	5.00E-13	-0.107168	0.0790466	0.175189
11	rs4731702	7	140174	C	T	0.512779	-0.0471297	0.00716051	9.20E-11	0.0660724	0.0632043	0.295856
12	rs55744247	5	641338	G	A	0.795882	-0.0534533	0.0088675	5.10E-10	-0.113855	0.0776773	0.142731
13	rs7538503	1	243742	A	G	0.710369	-0.0474118	0.00787047	8.40E-10	0.00713499	0.0695653	0.918309
14	rs8077609	17	190864	A	C	0.673982	0.0420664	0.00765749	1.10E-08	0.0845527	0.0676265	0.211204

Supplementary Table 7. Mendelian randomisation. Results for the association between body mass index (BMI), height adjusted gluteofemoral adipose tissue (GFAT), visceral adipose tissue (VAT), abdominal subcutaneous adipose tissue (ASAT) and Cardiovascular age-delta.

Outcome	Exposure	Method	Number of SNPs	Beta	Standard Error	*P* value
Cardiovascular age-delta	GFAT adjusted for BMI	MR Egger	25	-0.9174	0.8089	0.2684
Cardiovascular age-delta	GFAT adjusted for BMI	Weighted Median	25	-0.9183	0.3809	0.0159
Cardiovascular age-delta	GFAT adjusted for BMI	Inverse Variance Weighted	25	-0.9576	0.2877	0.0009
Cardiovascular age-delta	GFAT adjusted for BMI	Simple Mode	25	-0.4396	0.8312	0.6017
Cardiovascular age-delta	GFAT adjusted for BMI	Weighted Mode	25	-0.3853	0.8455	0.6527
Cardiovascular age-delta	VAT adjusted for BMI	MR Egger	17	1.1595	2.2425	0.6126
Cardiovascular age-delta	VAT adjusted for BMI	Weighted Median	17	-0.1102	0.4951	0.8238
Cardiovascular age-delta	VAT adjusted for BMI	Inverse Variance Weighted	17	0.3566	0.5343	0.5044
Cardiovascular age-delta	VAT adjusted for BMI	Simple Mode	17	-0.3572	0.8315	0.6733
Cardiovascular age-delta	VAT adjusted for BMI	Weighted Mode	17	-0.1982	0.8011	0.8078
Cardiovascular age-delta	ASAT adjusted for BMI	MR Egger	14	1.1547	2.0570	0.5849
Cardiovascular age-delta	ASAT adjusted for BMI	Weighted Median	14	0.4908	0.4533	0.2789
Cardiovascular age-delta	ASAT adjusted for BMI	Inverse Variance Weighted	14	0.6128	0.3656	0.0937
Cardiovascular age-delta	ASAT adjusted for BMI	Simple Mode	14	0.3370	0.8015	0.6810
Cardiovascular age-delta	ASAT adjusted for BMI	Weighted Mode	14	0.4573	0.7401	0.5473

Supplementary Table 8. Pairwise comparison between Group1 = Unfit and Group2 = Fit

This table shows the mean age-delta differences between "Unfit" and "Fit" participants by weight category. An adjusted *p*-value indicates whether the difference in mean age-delta between each pair of groups is statistically significant. The final two columns display the observed mean age-delta in Group1 and Group2, respectively.

group1	group2	Mean Diff	Adj. P-value	Lower 95% CI	Upper 95% CI	Mean age -delta (Group1)	Mean age -delta (Group2)
Unfit-Normal Weight	Fit-Normal Weight	-0.5768	0.0041	-1.0321	-0.1216	0.040317	-0.536495
Unfit-Normal Weight	Fit-Obese	-0.0945	0.9967	-0.6512	0.4622	0.040317	-0.054162
Unfit-Normal Weight	Fit-Overweight	0.1189	0.9755	-0.3327	0.5704	0.040317	0.159184
Unfit-Obese	Fit-Normal Weight	-0.4077	0.2444	-0.9393	0.1239	-0.128787	-0.536495
Unfit-Obese	Fit-Obese	-0.4100	0.0070	-0.9500	0.1300	0.990000	0.580000
Unfit-Obese	Fit-Overweight	0.2880	0.6297	-0.2405	0.8164	-0.128787	0.159184
Unfit-Overweight	Fit-Normal Weight	-1.1651	0.0000	-1.5969	-0.7333	0.628610	-0.536495
Unfit-Overweight	Fit-Obese	-0.6828	0.0040	-1.2205	-0.1451	0.628610	-0.054162
Unfit-Overweight	Fit-Overweight	-0.4694	0.0219	-0.8973	-0.0415	0.628610	0.159184

Supplementary Table 9. Subgroup Analysis of Age-Delta and Visceral Adipose Tissue by Fitness–Weight Category

For each combination of fitness level (Unfit or Fit) and weight status (Normal, Overweight, or Obese), we fit a model assessing how VAT (visceral adipose tissue volume) relates to cardiovascular age-delta. The table reports R^2, adjusted R^2, F-statistics (with associated p-values), and the VAT coefficient (Beta [95% CI], p) indicating how strongly VAT is associated with age-delta in each subgroup.

Subgroup	N	R^2	Adj R^2	F (p)	VAT (Beta [95% CI], p)
**Unfit-Normal Weight**	3411	0.015	0.013	6.883 (p=2.20e-06)	0.8280 [0.490, 1.166], p<0.001
**Unfit-Obese**	2390	0.031	0.028	9.788 (p=3.21e-09)	0.1543 [-0.074, 0.383], p=0.0185
**Unfit-Overweight**	2942	0.017	0.015	9.273 (p=9.06e-09)	0.4984 [0.286, 0.711], p<0.001
**Fit-Normal Weight**	4268	0.018	0.017	20.75 (p=1.33e-20)	0.8501 [0.642, 1.058], p<0.001
**Fit-Overweight**	5303	0.010	0.009	12.44 (p=4.90e-12)	0.4855 [0.341, 0.630], p<0.001
**Fit-Obese**	2927	0.036	0.034	19.08 (p=1.12e-18)	0.1949 [0.035, 0.355], p=0.017

Supplementary Table 10. Outcome analysis results. The table summarizes the associations between cardiovascular age-delta, adjusted for age and sex, and various cardiovascular outcomes, including all-cause mortality, composite cardiovascular outcomes (Major Adverse Cardiovascular Events, MACE), and individual conditions. Columns include estimated coefficients (coef), hazard ratios (exp(coef)), standard errors (se(coef)), 95% confidence intervals (coef lower and upper 95%, exp(coef) lower and upper 95%), z-statistics (z), P values (p), and -log2(p)

Outcome after MRI	coef	exp(coef)	se(coef)	coef lower 95%	coef upper 95%	exp(coef) lower 95%	exp(coef) upper 95%	z	p	-log2(p)
**Mortality Outcome**										
All-cause mortality	-0.16	0.86	0.14	-0.42	0.11	0.65	1.12	-1.14	0.26	1.97
**Composite Cardiovascular Outcome**										
MACE	-0.10	0.90	0.12	-0.33	0.12	0.72	1.13	-0.91	0.36	1.46
**Individual Cardiovascular Outcomes**										
Hypertension	0.11	1.11	0.06	-0.01	0.22	0.99	1.25	1.79	0.07	3.75
Atrial Fibrillation	0.15	1.16	0.06	0.02	0.27	1.02	1.31	2.24	0.02	5.33
Stroke	0.25	1.29	0.17	-0.07	0.58	0.93	1.78	1.51	0.13	2.93
Angina	0.08	1.09	0.07	-0.06	0.23	0.94	1.26	1.13	0.26	1.96
Heart Failure	0.02	1.02	0.08	-0.14	0.17	0.87	1.19	0.19	0.85	0.24
Myocardial Infarction	-0.16	0.86	0.17	-0.48	0.17	0.62	1.19	-0.94	0.35	1.52
Type 2 Diabetes	0.25	1.29	0.09	0.07	0.43	1.08	1.54	2.77	0.01	7.48

Supplementary Figures

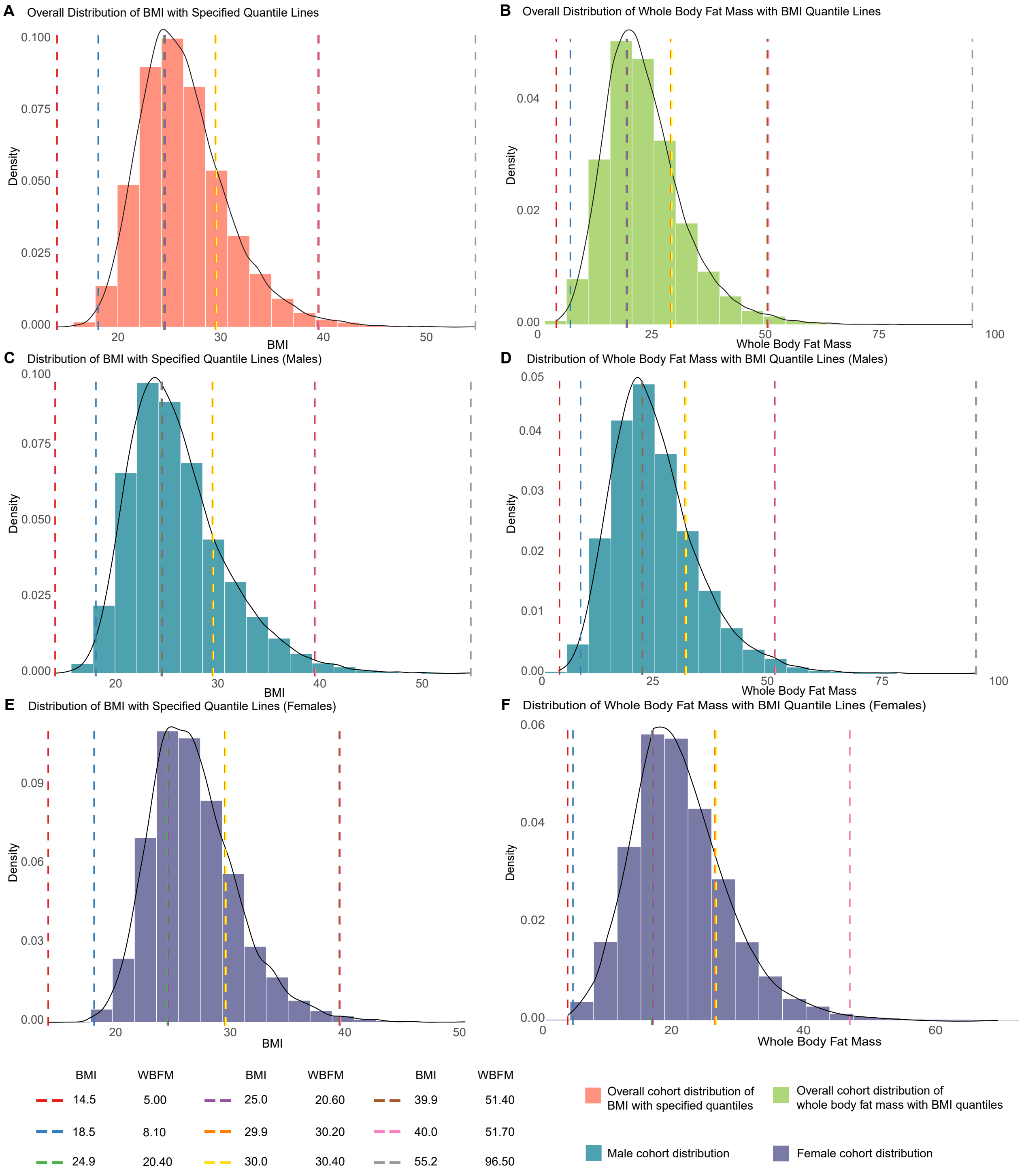

Supplementary Figure 1. Categorisation approach for whole body fat mass based on BMI quantiles. Density distributions of body mass index (BMI) and whole body fat mass (WBFM) for different cohorts with specified quantile lines; Panel **A** depicts the overall BMI distribution with quantile lines, showing the spread of BMI values in the entire cohort; Panel **B** illustrates the WBFM distribution for the entire cohort with BMI quantile lines; Panel **C** displays the BMI distribution for males with marked quantiles; Panel **D** represents the WBFM distribution for males, annotated with BMI quantiles; Panel **E** shows the BMI distribution for females similarly; Panel **F** demonstrates the WBFM distribution for females, indicating the density and quantile lines based on BMI. Quantile markers are provided for key percentiles, highlighting the central tendencies and distribution spread within each cohort.

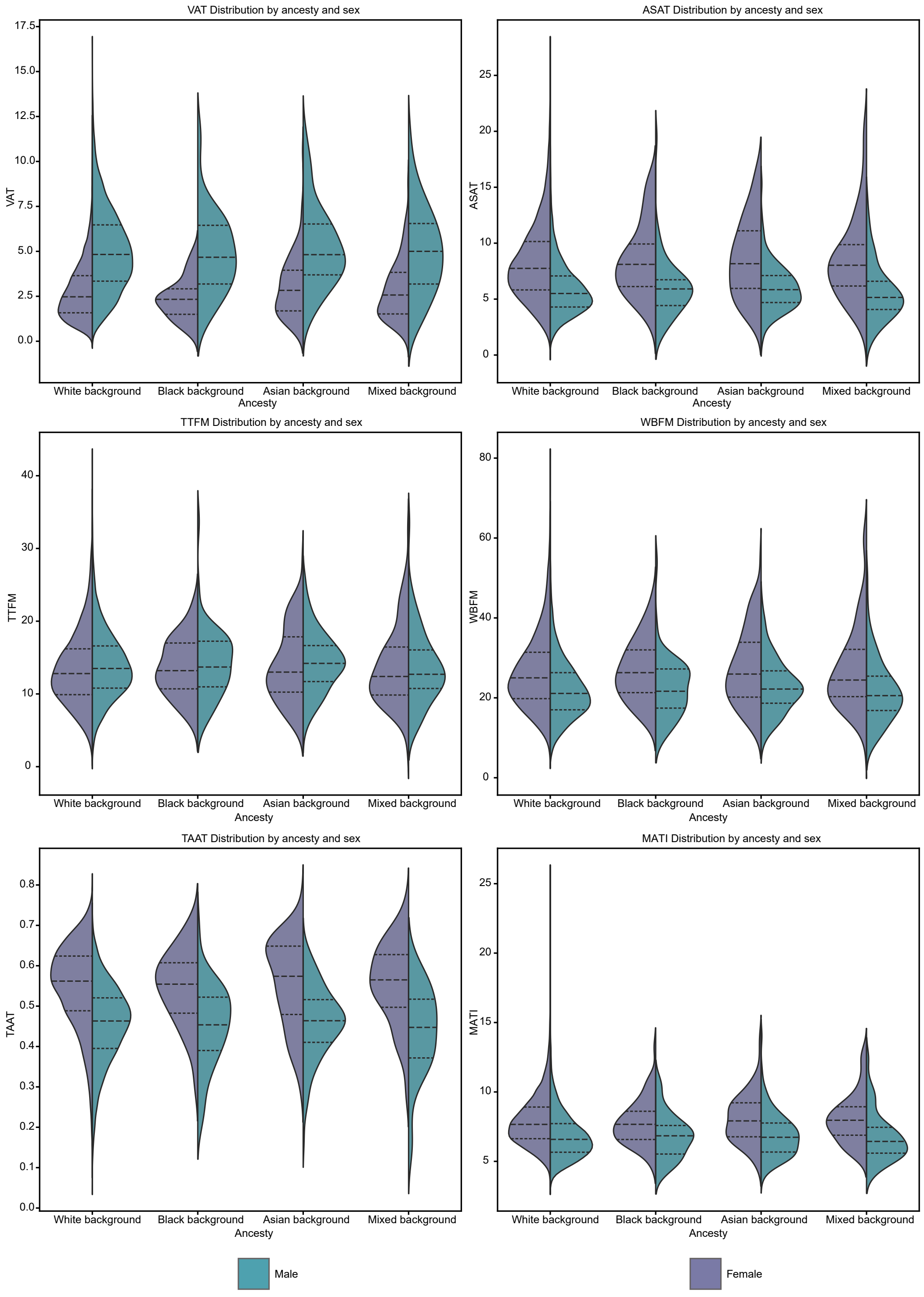

Supplementary Figure 2. Fat phenotypes distributions by ancestry. Violin plots show the distribution of fat phenotypes stratified by ancestry and sex: White background (n=20631, 97.12%), ; Black background (n=128, 0.60%); Asian background (n=262, 1.24%); Mixed background (n=220, 1.04%). Dashed lines show each quartile and values are shown in Supplementary Table 1. VAT, visceral adipose tissue; ASAT, abdominal subcutaneous adipose tissue; MATI, muscle adipose tissue infiltration; TTFM, total trunk fat mass; WBFM, whole body fat mass; TAAT, total abdominal adipose tissue.

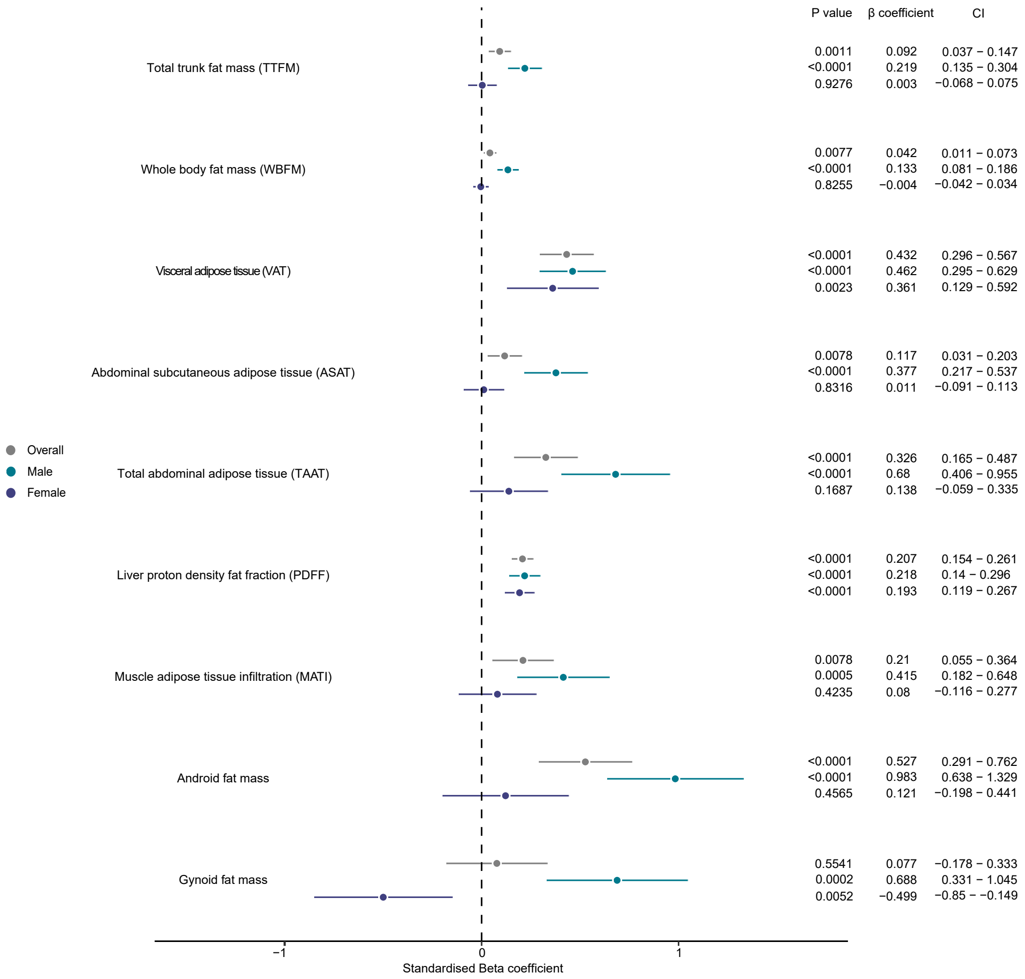

Supplementary Figure 3. Height^2-adjusted effect of fat phenotypes on cardiovascular age-delta. Linear regression analysis of quantitative adipose tissue traits (n=21,241 of which 5,168 had android and gynoid fat mass values) with height^2-adjusted cardiovascular age-delta as the dependent variable. *P* values, standardised beta-coefficient point estimates, and 95% confidence intervals are shown, stratified by sex. The adipose tissue traits include gynoid fat mass, android fat mass, muscle adipose tissue infiltration (MATI), liver proton density fat fraction (PDFF), total abdominal adipose tissue (TAAT), abdominal subcutaneous adipose tissue (ASAT), visceral adipose tissue (VAT), whole body fat mass (WBFM), and total trunk fat mass (TTFM)

.

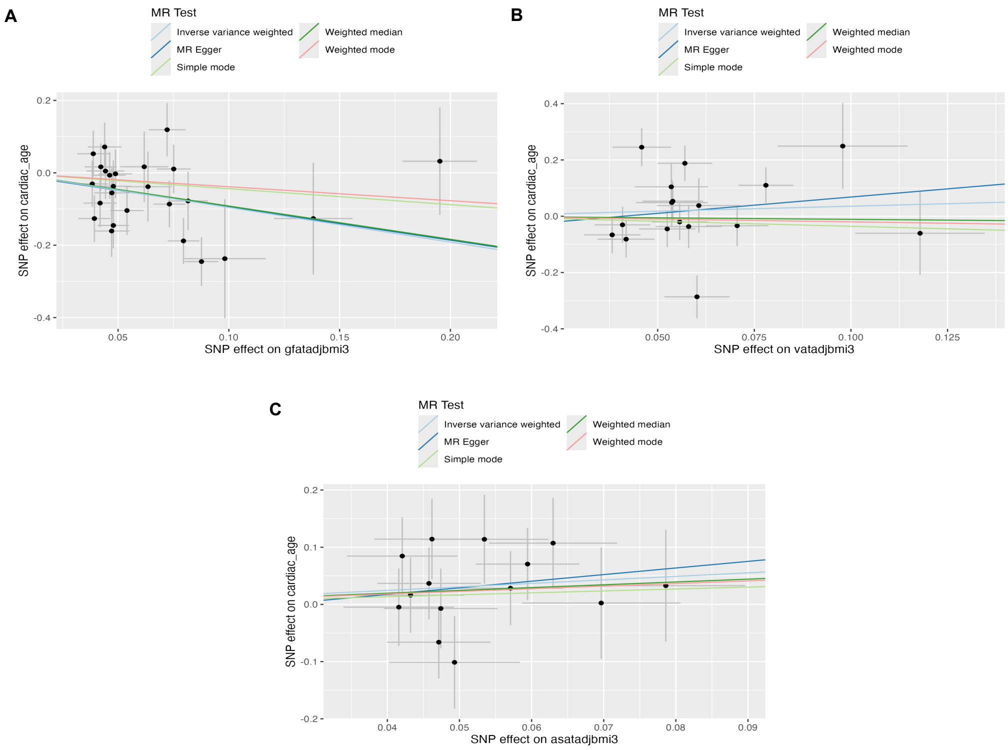

Supplementary Figure 4. Single nucleotide polymorphism (SNP) effects of body fat on cardiovascular age. Mendelian randomisation (MR) analysis of BMI adjusted **A** gluteofemoral adipose tissue, **B** visceral adipose tissue, and **C** abdominal subcutaneous adipose tissue as exposure, with cardiovascular age as outcome. Genetic instruments for body fat were selected from a published GWAS.[27] The effects (β) of the exposure variable-increasing allele at independent SNPs ($r^2 < 0.001$) reaching $P < 5e\text{-}8$ are plotted as datapoints and associated standard errors are represented as lines extending from datapoints. The plots were produced using the R package `TwoSampleMR`. See Supplementary Table 6 for full MR results and Supplementary Table 7 for full list of SNP IDs.

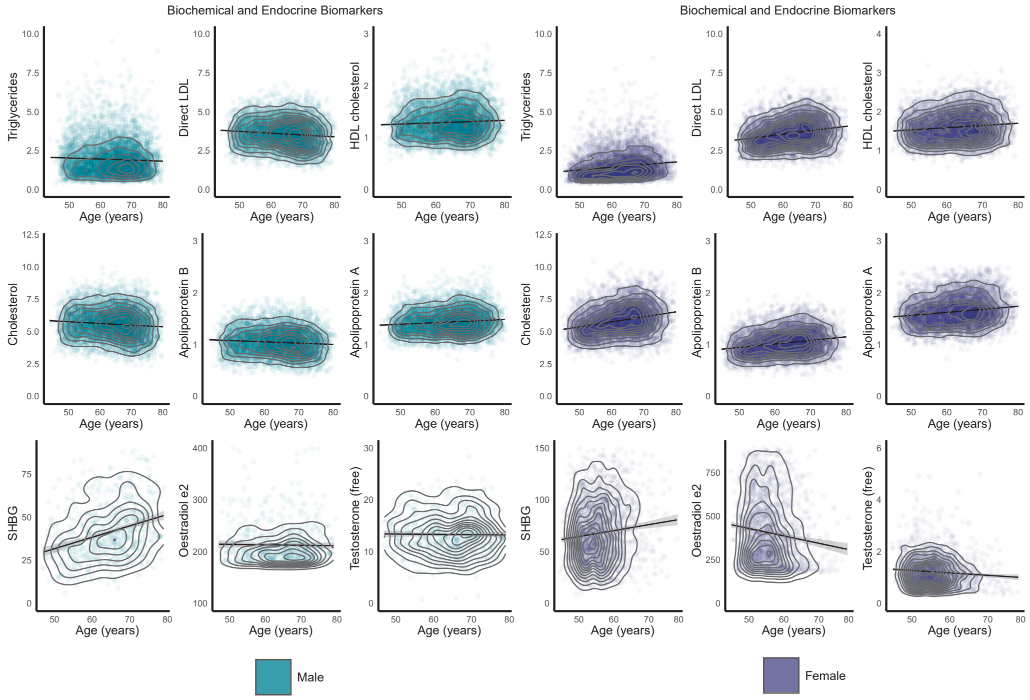

Supplementary Figure 5. Biomarker associations with chronological age. Circulating biomarkers (n=19,856) and hormones (n=3,588) are shown with their relationship to chronological age at the time of imaging (ages jittered, density contours, point colours represent coefficient of determination (R2). LDL, low density lipoprotein; HDL, high density lipoprotein; SHBG, sex hormone-binding globulin.
